# RG-SAPF: A Scheme for Cooperative Escorting of Underwater Moving Target by Multi-AUV Formation Systems Based on Rigidity Graph and Safe Artificial Potential Field

**DOI:** 10.3390/s25226823

**Published:** 2025-11-07

**Authors:** Wen Pang, Daqi Zhu, Mingzhi Chen, Wentao Xu

**Affiliations:** 1 School of Mechanical Engineering, University of Shanghai for Science and Technology, Shanghai 200093, China; wpang0708@usst.edu.cn (W.P.); mingzhichen@usst.edu.cn (M.C.); 2 School of Economics and Management, Shanghai Maritime University, Shanghai 201306, China; xuwentao1111@stu.shmtu.edu.cn

**Keywords:** underwater moving target, multi-AUV systems, formation control, target escort, path planning, safe artificial potential field

## Abstract

This paper addresses the challenge of cooperatively escorting a moving underwater target, such as a human-occupied vehicle (HOV), using a multi-AUV formation in complex ocean environments. We propose a comprehensive framework, RG-SAPF scheme, that integrates a rigidity graph (RG)-based reconfigurable formation control strategy with a safe artificial potential field (SAPF) motion planning method. The RG-based controller enables the AUVs to form and dynamically reconfigure a 3D escort formation around the target using only relative position information, ensuring the target remains within the formation’s convex hull. Meanwhile, the SAPF algorithm, enhanced with an adaptive Widrow–Hoff rule, enables real-time and collision-free path planning in obstacle-rich environments. Simulation and experimental results demonstrate that the proposed method effectively maintains formation integrity, supports flexible obstacle avoidance, and provides continuous target escort under dynamic conditions, validating its potential for practical underwater escort applications.

## 1. Introduction

Advancement in computer science, communication technology, underwater sensing, automatic control and underwater vehicle engineering have led to substantial development and widespread global adoption of automatic underwater vehicles (AUVs) over the past few decades [1,2,3]. Given their capacity to execute high-risk operations in challenging environments, it is foreseeable that AUVs will assume a more prominent role in future marine endeavours, including oceanographic surveys, exploration of marine resources, inspection of underwater cables, intelligence gathering, and surveillance of territorial seas. Separately, the development of the Human Occupied Vehicle (HOV) has reshaped the history of deep-sea exploration, playing a significant role in helping humanity to understand the ocean and develop marine resources [4]. However, the deep sea is characterised by extreme pressure, low temperatures, darkness and complex ocean currents. These harsh conditions pose a severe threat to the safety and stable operation of HOVs. Operating manned submersibles presents many challenges. To address these challenges and ensure the safety and effectiveness of HOVs during operations, the concept of a multi-AUV cooperative escort has emerged [5]. In this configuration, the AUVs are responsible for detecting underwater obstacles in the path of the research HOV and monitoring the underwater environment, e.g., water quality, temperature and current conditions. They can also provide early warnings of potential risks and be used as communication relays to enhance the connection between the HOV and the surface control centre. In contrast, HOVs focus exclusively on conducting core underwater missions. Due to their small size, ease of control and high intelligence, AUVs can maximise the safety of HOV personnel when exposed to external threats. The cooperation of an AUV fleet has attracted significant attention from scholars, compared with a single AUV. This is due to the fleet’s diverse potential application scenarios, scalability and robustness advantages that enable various marine tasks to be completed in complex environments while substantially improving operational efficiency. Based on the above analysis, HOVs carrying out missions under the escort of a fleet of cooperative AUVs are regarded as the framework for future underwater exploration, particularly in deep-sea environments [6].

AUV cooperative control research encompasses a broad range of topics, including collaborative search [7], collaborative capture [8], coordinated transportation [9], environmental monitoring [10], underwater communication networking [11], formation control [12] and path planning [13]. As a crucial branch of cooperative control, multi-AUV formation control aims to maintain a desired formation configuration while tracking a predefined trajectory. Due to its potential practical application value, this topic has received increasing attention, with numerous typical control algorithms having been developed by scholars and engineers, including the leader-follower method [14], the virtual structure-based approach [15], the consensus-based strategy [16], the behaviour based scheme [17], the graph theory-based technique [18], and the artificial potential field (APF)-based algorithm [19]. The leader–follower approach is widely adopted in traditional application scenarios. However, most of these studies have focused on specific behavioural processes, such as formation acquisition [20], formation maintenance [21] and reconfiguration [22], paying limited attention to the scenario of escorting mobile targets in seabed environments. The above-mentioned outstanding works have made their respective representative contributions in the fields of multi-robot cooperative control and formation control, which have greatly inspired us. But there is one common point: they seldom pay attention to the issue of escorting moving targets by agent formations.

**Figure 1:**
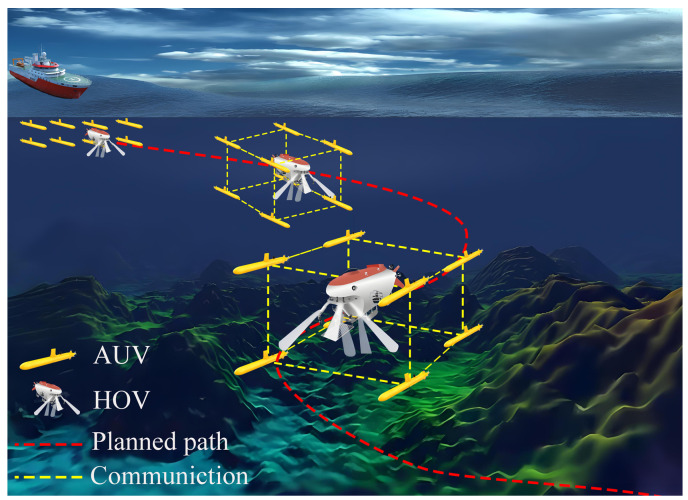
Example of a multi-AUV formation escort HOV in a complex obstacle environment.

As mentioned earlier, a typical example is the use of coordinated formation AUVs to escort moving targets, such as HOVs, or to track and monitor endangered species. As shown in Figure 1, this involves three essential technologies: (1) It involves initial escort formation acquisition and maintenance, which involves selecting and guiding multiple AUVs to the vicinity of the target in order to establish all-round encirclement. (2) It also involves planning a real-time maneuvering path for the moving target; the target then tracks the planned path in order to maneuver in the obstacle environment; the escort AUVs follow the target while maintaining the desired formation structure. (3) During movement, the escort formation AUVs must avoid dynamic and static obstacles by reconfiguring their formation in order to enhance adaptability to complex environments. During this process, the AUVs must continuously track, observe and escort the moving target.

Over the past few decades, research into formation escort for multi-agent systems (MASs) has been conducted in the domains of terrestrial, surface, and space applications. This has included scenarios involving unmanned ground vehicles (UGVs), unmanned surface vessels (USVs), and unmanned aerial vehicles (UAVs), with several notable achievements reported in the literature [23,24,25,26,27,28,29]. Work in [23] proposed a null space-based behavioural control approach to solving the moving target escort problem using a team of mobile robots. Unlike other existing behavioural coordination methods, this control strategy was validated through simulations and multiple experimental case studies. In [24], the authors proposed an end-to-end solution based on a deep reinforcement learning strategy to coordinate an escort team to protect high-value payloads. The author of [25] proposed a modelling method based on a signed graph to construct communication and coordination relationships among multiple USVs in order to solve the distributed leader escort control problem for USVs. Focusing on the economic benefits and personnel safety of merchant ship escort missions, ref. [26] addressed the challenges of USV formation mobility by analysing formation operation behaviours. Additionally, an obstacle avoidance behaviour function under a global fusion strategy was proposed based on the APF method to mitigate formation lag and trajectory oscillation. For UAVs, ref. [27] presented an autonomous cooperative flocking algorithm for heterogeneous UAV swarms to resolve the escort problem during group cruising. Meanwhile, ref. [28] proposed a flocking algorithm for networked miniature UAVs to execute aerial escort tasks. In this scenario, the following UAVs primarily rely on and adapt to the position of the target aerial vehicle in order to achieve time-varying formation escort flight. In [29], the authors used swarm intelligence and evolutionary optimisation to enable group self-organisation. They also employed a set of optimal parameters to study the cooperative control of a drone swarm that can surround and escort different types of targets. All of these studies, whether based on simulation or experimental verification, have produced excellent results. However, using multiple AUVs for the escort of dynamic underwater targets still presents many challenges.

1. Owing to the limitations of electromagnetic communication in underwater environments, only acoustic wave communication or laser communication can be adopted for underwater data transmission.
2. The underwater environment is far more complex than the terrestrial and aerial environments. This complexity is reflected not only in the challenges of seabed environment monitoring, such as seabed terrain scanning and moving obstacle perception, but also in the difficulties of controlling the three-dimensional (3D) topological movement of the escort formation.

Despite these challenges, research into the cooperative escort of underwater moving targets using multiple AUVs has gradually emerged in recent years. For example, in some oceanographic research projects, multiple underwater vehicles form formations to escort research vessels. In the military, multiple underwater vehicle formations are sometimes used to escort submarines. Many scholars have conducted in-depth research in this area. Specifically, ref. [30] proposed a cooperative control-based underwater target escort mechanism which integrates a belief function method-based self-organising map algorithm for task assignment and an APF-based formation control method. This mechanism enables the escort of a multi-AUV formation during target maneuvering while achieving obstacle avoidance. Furthermore, ref. [5] presented an artificial flow potential field algorithm combining the APF approach with the gradient descent algorithm to plan paths for multi-submersible systems. To enhance the safety and efficiency of path planning, the authors proposed an obstacle avoidance mechanism for escort formations based on a dual leader–follower algorithm, capable of addressing various scenarios. However, neither of these studies considered the presence of dynamic obstacles. Another study [31] established a distributed time-varying optimisation problem model for multi-AUV cooperative escort scenarios and employed a distributed fixed-time neurodynamic algorithm to solve it. Simulation results verified the effectiveness and stability of the designed algorithm for target tracking, formation control and dynamic obstacle avoidance. These excellent results are highly relevant to research on multi-underwater vehicle formation escort.

However, although progress has been made in the application of multi-AUV formations to escort missions, notable limitations remain. A key challenge lies in their adaptability to complex environments; the underwater environment is inherently complex and fraught with uncertainties. Consequently, path planning must consider as many influencing factors as possible, such as external ocean current interference and static and dynamic obstacles, in order to prevent overall path deviation and ensure that the submersibles reach their designated positions. Simultaneously, formation control and obstacle avoidance issues require further attention. Two critical considerations are required: firstly, feasible movement paths must be planned for the formation in real time, and secondly, the reconfigurability of the formation must be prioritised while ensuring the escort AUVs achieve all-round, real-time envelopment of the target. Addressing these issues is crucial for the further development and widespread application of multi-AUV formation escort technology.

The algorithms currently employed for AUV underwater path planning can be categorised as either global or local trajectory planning algorithms. The former includes two subcategories: (1) traditional algorithms such as Dijkstra’s algorithm, Rapidly-exploring Random Tree (RRT), Probabilistic Road Map (PRM) and Dubins curves and (2) intelligent algorithms such as Particle Swarm Optimization (PSO), A*, Salp Swarm Algorithm (SSA), Artificial Bee Colony (ABC), Neural Network (NN) and Reinforcement Learning (RL)-based algorithms [32,33,34]. The latter mainly comprises the APF method, Model Predictive Control (MPC), Mathematical Optimisation Algorithm (MOA), Dynamic Window Approach (DWA) and Bug algorithms [35,36]. Compared with other algorithms, the APF-based method offers simpler mathematical modelling and implementation. Due to these advantages, APF-based methods have been widely adopted for path planning and target searching tasks in intelligent robots, including AUVs.

Building on prior studies, this paper proposes multi-AUV formation control technology and a formation-based escort mechanism for moving targets. Specifically, the formation control technology uses an undirected rigid graph (RG) to constrain the distances between the AUVs and between the AUVs and the target. It achieves target envelopment via reverse step control technology. Meanwhile, the escort mechanism plans the maneuvering path of the escort formation using a safe artificial potential field (SAPF) algorithm. Collectively, this integrated mechanism encompasses three core schemes: escort formation control, trajectory planning and formation reconfiguration. Regarding formation control, the proposed RG-based formation control method draws significant inspiration from [37,38,39]. In these studies, researchers developed a rigid graph-based formation control method that uses distance to guide multiple robots to specified positions. However, the existing method has limitations, such as an inability to reconfigure formations and insufficient flexibility in obstacle avoidance. These limitations are addressed by the method proposed in this paper. For formation motion planning, an adaptive path strategy inspired by [40] was designed by integrating the SAPF algorithm and Widrow–Hoff rules. This strategy enables formations to manoeuvre smoothly and accurately reach target positions without colliding with obstacles. Regarding formation reconfiguration, time-varying reconfiguration of the escort formation configuration is realised based on an affine transformation. The overall objective of the proposed technology and mechanism is to maintain a stable three-dimensional escort formation while AUVs follow and escort moving targets, enabling flexible obstacle avoidance.

Leveraging rigid graph theory enables submersibles to form and maintain a formation with minimal inter-vehicle communication under constrained underwater positioning and communication conditions, relying solely on the relative velocities and distances between neighbouring submersibles. The incorporation of Lyapunov function constraints mathematically guarantees the recursive feasibility and closed-loop stability of the algorithm. On the other hand, the APF algorithm is simple in principle, has a small computational load, and a fast response speed. It can provide a relatively short, locally minimal, and collision-free smooth path that bypasses obstacles, making it suitable for the rapid response of HOVs in complex underwater environments. Therefore, we plan the motion path for the submersible based on the modified APF algorithm.

This study makes significant advancements in the field of cooperative escort for underwater moving targets using multi-AUV formations, with the following key contributions:

1. Proposed the RG-SAPF comprehensive target escort mechanism: To enhance the practical applicability of multi-AUV cooperative systems in underwater target escort scenarios, this research integrates rigid graph (RG) theory, a safe artificial potential field (SAPF) algorithm, and the Widrow–Hoff rule. This integrated mechanism addresses three core challenges in escort missions: formation acquisition and maintenance, real-time collision-free path planning, and flexible formation reconfiguration, providing a systematic solution for multi-AUV cooperative escort tasks.
2. Developed a novel RG-based formation control and affine transformation-driven reconfiguration method: Combines rigidity graph (RG)-based formation design with affine transformation-based reconfiguration. This approach enables AUV formations to be reconfigured using only relative position information, with minimal inter-vehicle communication. The method ensures that the moving target remains at the geometric center of the formation at all times, thereby improving monitoring coverage and formation stability during escort missions.
3. Designed an adaptive path planning algorithm integrating SAPF and the Widrow–Hoff rule: To overcome the limitations of traditional artificial potential field (APF) methods, such as the “Goal Non-reachable With Obstacle Nearby (GNWON)” problem and excessive initial attractive force, a new adaptive path planning algorithm is designed by integrating the APF with the Widrow–Hoff rule. This algorithm enables real-time and flexible route planning for the formation, effectively addressing issues such as local minima and goal non-reachability in the presence of obstacles (GNWON).

The remainder of this paper is structured as follows: Section 2 introduces the AUV model, the rigid graph theory, the classical APF method, and formulating the research problem. Section 3 presents the main result of adaptive safe APF. The technical implementation of the formation control is presented in Section 4. Section 5 analyzes the performance using simulations. Section 6 reports the experimental results. Finally, Section 7 concludes the paper.

## 2. Preliminaries and Problem Formulation

### 2.1. Rigid Graph Theory

The control algorithm proposed in this paper is based on fundamental concepts from rigid graph theory, which are explained in detail in [41]. Specifically, the escort formation structure and communication links of the submersibles can be modelled using an undirected graph, denoted as G=(V,E,A), where V={1,2,…,n} represents the set of vertices (with *n* denoting the number of submersibles), and E⊂V×V denotes the set of undirected edges connecting these vertices. If vertex i∈V can receive information from the vertex j∈V, then there must be an edge between vertices *i* and *j*, i.e., (i,j)∈E, in which case vertices *i* and *j* are defined as adjacent. The adjacency matrix associated with the graph G is represented by $A=\left[a_{ij}\right]\in R^{n\times n}$, where $a_{ij}$ indicates the presence of an undirected connection between vertices *i* and *j*. If (i,j)∈E, then $a_{ij}=1$; otherwise, $a_{ij}=0$. Specifically, $a_{ii}=0$. The neighbours of a vertex refer to the vertices from which it can acquire information, the set of neighbors of vertex *i* is denoted by $N_{i}\left(E\right)=\left\{j\in V|(i,j)\in E\right\}$. $l\in \left\{1,2,\ldots ,n(n-1)/2\right\}$ yields the number of edges in the graph structure [37]. Let $p_{i}\in R^{3}$ represent the coordinates of vertex *i*, and $p=\left[p_{1},p_{2},\ldots ,p_{n}\right]\in R^{3n}$ represents the set of all vertex coordinates. The formation framework F=(G,p) is then defined as a realization of graph G in 3D space, determined by the given point set *p*. The edge function $f_{G}\left(p\right):R^{3n}\to R^{l}$ of (G,p) is expressed as

(1) $$f_{G}\left(p\right)=\left(\ldots ,{∥p_{i}-p_{j}∥}^{2},\ldots \right),\left(i,j\right)\in E$$

where $∥\cdot ∥$ denotes the Euclidean norm, and each element given by ${∥p_{i}-p_{j}∥}^{2}$ corresponds to an edge in E that connects the *i*th and *j*th vertices. Based on the edge function, the rigidity matrix $R\left(p\right):R^{3n}\to R^{l\times 3n}$ of the formation framework (G,p) can be defined as follows:

(2) $$R\left(p\right)=\frac{1}{2}\frac{\partial f_{G}\left(p\right)}{\partial p}$$

It is well known that a graph G is minimally rigid in 3D space if and only if $\left|E\right|=3\left|V\right|-6$ [42]. For a minimally rigid graph, its corresponding rigidity matrix R(p) has full row rank. If the transpose matrix $R^{T}\left(p\right)$ has full column rank and $\mathrm{rank}\left[R\left(p\right)\right]=\mathrm{rank}\left[R\left(p\right)R^{T}\left(p\right)\right]$, then $R\left(p\right)R^{T}\left(p\right)\in R^{\left|E\right|\times \left|E\right|}$ is invertible [42].

**Lemma** **1** ([37])**.** *Let $\mu \left(t\right)\in R^{3}$, and $1_{n}$ denotes the n-dimensional vector of ones; then, $R\left(p\right)\left(1_{n}⊗\mu \right)=0$.*

### 2.2. Submersible Model

To facilitate the analysis of collaborative target escort formation control and motion planning for multi-submersible systems, the following assumptions are proposed to clarify the controller design.

**Assumption** **1.** *In a multi-submersible formation escort system, each submersible is equipped with a suite of reliable navigation sensors, including sonar, an inertial navigation system (INS), an ultra-short baseline (USBL) positioning system, an underwater vision camera, and an altimeter. Integrating of these multi-sensor navigation components enables each submersible to achieve high-precision navigation and effectively detect of underwater obstacles.*

**Assumption** **2.** *The multi-submersible formation system uses underwater acoustic communication, to allow the *i*th submersible (i∈V) to exchange information with its neighbouring submersibles.*

**Figure 2:**
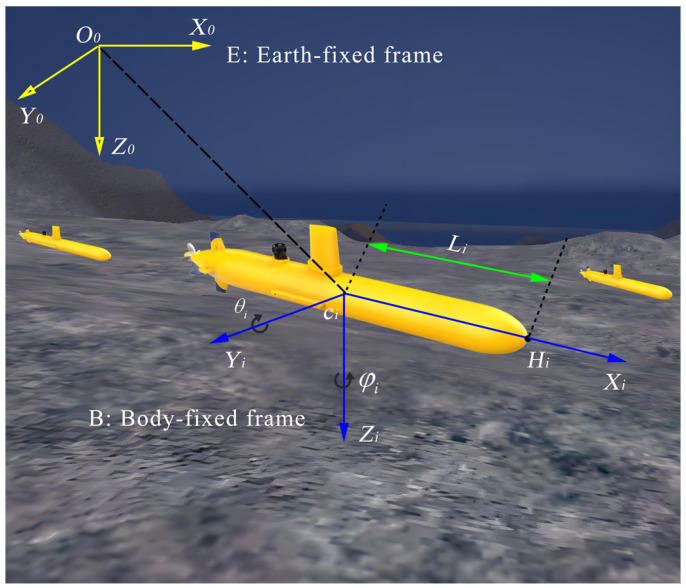
The 3D configuration of *i*th escort AUV.

This study considers a 3D escort formation system comprising multiple submersibles, including one HOV and several AUVs. The Figure 2 illustrates the configuration of the *i*th AUV, where $\left\{E\right\}=\left\{O_{0}-X_{0},Y_{0},Z_{0}\right\}$ denotes the Earth-fixed frame and $\left\{B\right\}=\left\{O_{i}-X_{i},Y_{i},Z_{i}\right\}$ denotes the body-fixed frame. The kinematic and dynamic models of each AUV are then described as follows [37,38].

(3a) $${\dot{p}}_{ci}=S\left(\theta_{i},\varphi_{i}\right)\eta_{i}$$

(3b) $${\bar{M}}_{i}{\dot{\eta }}_{i}+{\bar{D}}_{i}\eta_{i}={\bar{\tau }}_{i}$$

in which, $p_{ci}={\left[x_{ci},y_{ci},z_{ci},\theta_{i},\varphi_{i}\right]}^{T}$ represents the position of the mass point $\left(x_{ci},y_{ci},z_{ci}\right)$ of the *i*th escort AUV and its heading angles $\left(\theta_{i},\varphi_{i}\right)$ relative to the frame {E}. The vector $\eta_{i}={\left[v_{i},w_{\theta i},w_{\varphi i}\right]}^{T}\in R^{3}$ denotes the generalized velocity of the *i*th escort AUV, where $v_{i}$ is the linear velocity along the x-axis of the body-fixed frame {B}, and $w_{\theta i}$, $w_{\varphi i}$ denote the angular velocities about the *z*-axis and *x*-axis of {B}, respectively. $S(\theta_{i},\varphi_{i})$ is the transformation matrix (mapping body-fixed velocities to earth-fixed velocities) and is defined as

(4) $$S\left(\theta_{i},\varphi_{i}\right)=\left[\begin{array}{ccc} c\theta_{i}c\varphi_{i} & 0 & 0\\ c\theta_{i}s\varphi_{i} & 0 & 0\\ s\theta_{i} & 0 & 0\\ 0 & 1 & 0\\ 0 & 0 & 1 \end{array}\right]$$

where $c\theta_{i}\overset{\Delta }{=}\cos\theta_{i}$, $s\theta_{i}\overset{\Delta }{=}\sin\theta_{i}$, $c\varphi_{i}\overset{\Delta }{=}\cos\varphi_{i}$ and $s\varphi_{i}\overset{\Delta }{=}\sin\varphi_{i}$, respectively. In (3b), ${\bar{M}}_{i}=\mathrm{diag}\left\{m_{i},I_{1i},I_{2i}\right\}$ represents the inertia matrix of the *i*th AUV, where $m_{i}$ is the mass of the *i*th AUV and $I_{1i}$, $I_{2i}$ denote its moment of inertia about the *y*- and *z*-axes of the body-fixed frame {B}, respectively. ${\bar{D}}_{i}\in R^{3\times 3}$ denotes the constant damping matrix (including both linear and quadratic damping effects), and ${\bar{\tau }}_{i}\in R^{3}$ is the force/torque-level control input of the *i*th AUV. From (3a), it can be observed that the motion constraint space ($\eta_{i}\in R^{3}$) has a lower dimensionality than the system state space ($p_{ci}\in R^{5}$). To address the nonholonomic constraints and dynamic model constraints of AUVs, we adopt the approach proposed in [38,39], and define the “hand position” $p_{i}={\left[x_{i},y_{i},z_{i}\right]}^{T}\in R^{3}$ for the *i*th AUV, as illustrated in Figure 2.

(5) $$p_{i}=\left[\begin{array}{c} x_{i}\\ y_{i}\\ z_{i} \end{array}\right]=\left[\begin{array}{c} x_{ci}\\ y_{ci}\\ z_{ci} \end{array}\right]+L_{i}\left[\begin{array}{c} \cos\theta_{i}\cos\varphi_{i}\\ \cos\theta_{i}\sin\varphi_{i}\\ \sin\theta_{i} \end{array}\right]$$

where $L_{i}$ denotes the distance from the center of mass ($c_{i}$) of the *i*th AUV to the defined point $H_{i}$, as illustrated in Figure 2. Then, by taking the first-order derivative of (5) with respect to time, the following expression is obtained:

(6) $${\dot{p}}_{i}=\left[\begin{array}{ccc} c\theta_{i}c\varphi_{i} & -L_{i}c\theta_{i}s\varphi_{i} & -L_{i}s\theta_{i}c\varphi_{i}\\ c\theta_{i}s\varphi_{i} & L_{i}c\theta_{i}c\varphi_{i} & -L_{i}s\theta_{i}c\varphi_{i}\\ s\theta_{i} & 0 & L_{i}c\theta_{i} \end{array}\right]\eta_{i}$$

Then, based on Equations (3a), (3b) and (4), through some mathematical transformations, we have

(7) $$\eta_{i}=J\left(\theta_{i},\varphi_{i}\right){\dot{p}}_{i}$$

where

$$J\left(\theta_{i},\varphi_{i}\right)=\left[\begin{array}{ccc} c\theta_{i}c\varphi_{i} & c\theta_{i}c\varphi_{i} & s\theta_{i}\\ -s\varphi_{i}/L_{i}c\theta_{i} & c\varphi_{i}/L_{i}c\theta_{i} & 0\\ -s\theta_{i}c\varphi_{i}/L_{i} & -s\theta_{i}s\varphi_{i}/L_{i} & c\theta_{i}/L_{i} \end{array}\right]$$

By taking the time derivative of (7), we obtain ${\dot{\eta }}_{i}=\dot{J}\left(\theta_{i},\varphi_{i}\right){\dot{p}}_{i}+J\left(\theta_{i},\varphi_{i}\right){\ddot{p}}_{i}$. Subsequently, pre-multiplying both sides of this resulting equation by matrix ${\bar{M}}_{i}$ and substituting (3b) into the expression yields

(8) $${\bar{M}}_{i}\dot{J}\left(\theta_{i},\varphi_{i}\right){\dot{p}}_{i}+{\bar{M}}_{i}J\left(\theta_{i},\varphi_{i}\right){\ddot{p}}_{i}+{\bar{D}}_{i}J\left(\theta_{i},\varphi_{i}\right){\dot{p}}_{i}={\bar{\tau }}_{i}$$

Next, pre-multiply both sides of (8) by $J^{T}\left(\theta_{i},\varphi_{i}\right)$ yields the following Euler–Lagrange-like dynamic model

(9) $$M_{i}\left(p_{i}\right){\ddot{p}}_{i}+C_{i}\left(p_{i},{\dot{p}}_{i}\right){\dot{p}}_{i}+D_{i}\left(p_{i}\right){\dot{p}}_{i}=\tau_{i}$$

where

$$M_{i}\left(p_{i}\right)=J^{T}{\bar{M}}_{i}J,C_{i}\left(p_{i},{\dot{p}}_{i}\right)=J^{T}{\bar{M}}_{i}\dot{J},$$

$$D_{i}\left(p_{i}\right)=J^{T}{\bar{D}}_{i}J,\tau_{i}=J^{T}{\bar{\tau }}_{i}$$

where $p_{i}\in R^{3}$ denotes the generalized position of the *i*th AUV, $M_{i}\left(p_{i}\right)\in R^{3\times 3}$ is a positive-definite inertia matrix, $C_{i}\left(p_{i},{\dot{p}}_{i}\right)\in R^{3\times 3}$ stands for the Coriolis and centripetal matrix, $D_{i}\left(p_{i}\right)\in R^{3\times 3}$ is the damping matrix (incorporating frictional effects), and $\tau_{i}\in R^{3}$ denotes the control input vector.

For the Euler–Lagrange system described by (9), three key properties are commonly cited in [37,43], which are critical to the subsequent controller design and stability analysis. Property 1 ensures the controllability of the inertia matrix of the system. Property 2 is used to simplify the Lyapunov stability analysis, while Property 3 enables the system to be dynamically linearly parameterised, which facilitates the design of adaptive controllers. Together, these properties form the basis for subsequent controller design and stability proof. These properties are summarized as follows

**Property** **1.** *The inertia matrix $M_{i}\left(p_{i}\right)$ is symmetric, bounded, and uniformly positive definite.*

**Property** **2.** *For any arbitrary vector $\mu \in R^{3}$, the matrices $M_{i}\left(p_{i}\right)$ and $C_{i}\left(p_{i},{\dot{p}}_{i}\right)$ satisfy $\mu^{T}\left(M_{i}\left(p_{i}\right)-2C_{i}\left(p_{i},{\dot{p}}_{i}\right)\right)\mu =0$.*

**Property** **3.** *Each system in (9) with parametric uncertainties can be rearranged into the following linear parameterization form*

(10) $$M_{i}\left(p_{i}\right){\dot{\mu }}_{i}+C_{i}\left(p_{i},{\dot{p}}_{i}\right)\mu_{i}+D_{i}\left(p_{i}\right){\dot{p}}_{i}=Y_{i}\left(p_{i},{\dot{p}}_{i},\mu_{i},{\dot{\mu }}_{i}\right)\phi_{i}$$

where $Y_{i}\left(p_{i},{\dot{p}}_{i},\mu_{i},{\dot{\mu }}_{i}\right)\in R^{3\times 12}$ is a known regression matrix, and $\phi_{i}\in R^{12}$ denotes a vector of unknown but constant dynamic parameters. These parameters include a series of physical properties associated with the *i*th AUV, specifically given as

$$\phi_{i}={\left[\begin{array}{c} {\left[\begin{array}{cccc} m_{i} & I_{1i}/L_{i}^{2} & I_{2i}/L_{i}^{2} & {\left({\bar{D}}_{i}\right)}_{11} \end{array}\right]}^{T}\\ {\left[\begin{array}{cccc} {\left({\bar{D}}_{i}\right)}_{12}/L_{i} & {\left({\bar{D}}_{i}\right)}_{13}/L_{i} & {\left({\bar{D}}_{i}\right)}_{21}/L_{i} & {\left({\bar{D}}_{i}\right)}_{22}/L_{i}^{2} \end{array}\right]}^{T}\\ {\left[\begin{array}{cccc} {\left({\bar{D}}_{i}\right)}_{23}/L_{i}^{2} & {\left({\bar{D}}_{i}\right)}_{31}/L_{i} & {\left({\bar{D}}_{i}\right)}_{32}/L_{i}^{2} & {\left({\bar{D}}_{i}\right)}_{33}/L_{i}^{2} \end{array}\right]}^{T} \end{array}\right]}^{T}$$

### 2.3. Traditional Artificial Potential Field

The APF based path planning approach assumes that a vehicle, denoted as *q*, moves within an abstract artificial force field comprising two components: an attractive potential field and a repulsive potential field [44]. The attractive field is generated by the goal and directs the agent towards the goal position. By contrast, the repulsive field is formed by the superposition of repulsive effects from various obstacles, oriented away from them. Under the combined influence of these two fields, the agent moves from regions of high potential energy to regions of low potential energy, following the negative gradient of the total potential energy field. The goal position is typically designed to be the global minimum of the potential field, which theoretically ensures that the agent will eventually arrive at this position. The APF algorithm has several advantages: its simple structure, high computational efficiency, suitability for real-time path planning and the fact that it produces smooth paths without the need for secondary smoothing processing. Numerous potential functions have been proposed in the academic literature to model these effects [45].

The most commonly used attractive potential function is

(11) $$U_{att}^{*}\left(q\right)=\frac{1}{2}k_{att}d^{2}\left(q,p_{g}\right)$$

where $k_{att}$ is a positive scaling factor (adjusting the strength of the attractive effect), $d(q,p_{g})$ is the distance between the vehicle *q* and the goal $p_{g}$.

The attractive force is given by the negative gradient of the attractive potential

(12) $$F_{att}^{*}\left(q\right)=-\nabla U_{att}^{*}\left(q\right)=-k_{att}d\left(q,p_{g}\right)\cdot \frac{\partial d(q,p_{g})}{\partial (q)}$$

The attractive force tends to zero as the vehicle closes to the goal. The traditional repulsive potential function is

(13) $$U_{rep}^{*}\left(q\right)=\left\{\begin{array}{cc} \frac{1}{2}k_{rep}{\left(\frac{1}{d(q,p_{obs})}-\frac{1}{\rho_{0}}\right)}^{2}, & d\left(q,p_{obs}\right)\leq \rho_{0}\\ 0, & d\left(q,p_{obs}\right)>\rho_{0} \end{array}\right.$$

where $k_{rep}$ is a positive scaling factor (adjusting the strength of the repulsive effect), $d(q,p_{obs})$ represents the shortest distance from the vehicle *q* to the obstacle, $\rho_{0}$ denotes the maximum influence distance of the obstacle (i.e., the repulsive potential field only acts when the vehicle is within $\rho_{0}$ of the obstacle). The negative gradient of this repulsive potential function, which corresponds to the repulsive force acting on the vehicle and is given by

(14) $$\begin{array}{c} F_{rep}^{*}\left(q\right)=-U_{rep}^{*}\left(q\right)=\left\{\begin{array}{cc} k_{rep}\left(\frac{1}{d(q,p_{obs})}-\frac{1}{\rho_{0}}\right)\frac{1}{d^{2}\left(q,p_{obs}\right)}\cdot \frac{\partial d(q,p_{obs})}{\partial (q)}, & d\left(q,p_{obs}\right)\leq \rho_{0}\\ 0, & d\left(q,p_{obs}\right)>\rho_{0} \end{array}\right. \end{array}$$

The traditional total potential energy and resultant potential force acting on the vehicle *q* are $U_{total}^{*}\left(q\right)=U_{att}^{*}\left(q\right)+U_{rep}^{*}\left(q\right)$ and $F_{total}^{*}\left(q\right)=F_{att}^{*}\left(q\right)+F_{rep}^{*}\left(q\right)$, respectively. These determine the motion direction of the vehicle.

### 2.4. Problem Formulation

This study focuses on the escort formation maneuvering and obstacle avoidance problems of multi-submersible systems under two constraints: unknown absolute positions and limited communication. The escort AUV can only obtain its attitude information and the relative distances and velocities of neighbouring submersibles. To describe the desired escort formation shape, an infinitesimally and minimally rigid framework $F^{*}=\left(G^{*},p^{*}\right)$ is adopted, where $G^{*}=\left(V^{*},E^{*},A^{*}\right)$ (with $\dim(G^{*})=n$ represents the number of submersibles in the formation, $\dim(E^{*})=l$ not only represents the minimum number of edges required to stabilize the formation but also corresponds to the minimum number of communications links needed between submersibles), and $p^{*}=\left[p_{1}^{*},\ldots ,p_{n}^{*}\right]$, denoting the set of desired positions for each submersibles. For any two submersibles i,j∈V, the time-varying desired distance can be denoted by

(15) $$d_{ij}=∥p_{i}^{*}-p_{j}^{*}∥>d_{\mathrm{safe}}$$

where $d_{\mathrm{safe}}$ denotes the minimum safe distance between submersibles in the escort formation.

The actual escort formation shape of the submersibles in the escort can also be characterized by an infinitesimally rigid framework F=(G,p), where $p=[p_{1},\ldots ,p_{n}]$ represents the set of the submersibles’ actual positions in real-time. It is assumed that the relative position, $p_{i}-p_{j}$, between each pair of submersibles in the framework F is measurable. Furthermore, at the initial time t=0, the submersibles do not satisfy the desired inter-submersible distance constraints, that is to say, $∥p_{i}\left(0\right)-p_{j}\left(0\right)∥\neq d_{ij}$ for all $i,j\in V^{*}$.

This study addresses two core control problems for the multi-submersible system: escort formation maneuvering and formation obstacle avoidance. A common primary control objective for both problems is to design the control input $\tau_{i}=\tau_{i}\left(p_{i},p_{i}-p_{j},v_{i},v_{i}-v_{j},d_{ij},{\dot{d}}_{ij}\right),i=1,2,\ldots ,n$, where $j\in N_{i}\left(E^{*}\right)$ is the neighbour of the *i*th submersible, such that

(16) $$∥p_{i}\left(t\right)-p_{j}\left(t\right)∥\to d_{ij}\left(t\right)\;\mathrm{as}\;t\to \infty ,\left(i,j\right)\in E^{*}$$

For the formation maneuvering problem, the secondary control objective is given by

(17) $${\dot{p}}_{i}\left(t\right)-v_{d}\left(t\right)\to 0\;\mathrm{as}\;t\to \infty ,i=1,2,\ldots ,n$$

where $v_{d}\in R^{3}$ represents the intended escort navigation speed of the submersible formation (a bounded, continuous function), and this speed is known to all submersibles. The control design is based on the assumption that the dynamic parameter vector $\phi_{i}$ in (10) is unknown. To address this, we define the parameter estimation error for the *i*th submersible as

(18) $${\tilde{\phi }}_{i}={\hat{\phi }}_{d}-\phi_{i}$$

where ${\hat{\phi }}_{i}\left(t\right)$ represents the dynamic estimate of $\phi_{i}$, which will be designed in the adaptive control framework.

In the trajectory tracking and obstacle avoidance problem, we designate the nth submersible (HOV) as the leader, with the remaining escort AUVs serving as followers. Our control protocol comprises three components: (a) Selecting the desired formation framework $F^{*}$ such that $p_{n}^{*}\in \mathrm{conv}\left\{p_{1}^{*},\ldots ,p_{n-1}^{*}\right\}$ lies in the convex hull of the predefined region (where convex hull is represented by conv{·}). This involves forming an escort convoy of AUVs surrounding the HOV, (b) enabling the leader HOV to track the pre-planned trajectory waypoint $p_{t}$ and avoid obstacle in a cluttered environment, and (c) ensuring the follower AUVs track the leader HOV while maintaining the desired time-varying formation. Therefore, the secondary control objective for the escort formation trajectory tracking and obstacle avoidance problem can be summarized as follows [46]

(19) $$p_{t}\left(t\right)\in \mathrm{conv}\left\{p_{1}\left(t\right),p_{2}\left(t\right),\ldots ,p_{n}\left(t\right)\right\}\;\mathrm{as}\;t\to \infty$$

where $p_{t}\in R^{3}$ represents the position of the planned trajectory waypoint for the leader HOV. We assume that the planned waypoint $p_{t}\left(t\right)$ satisfies two conditions: first, its third-order derivative is continuous, and secondly, $p_{t}$ and all its derivatives are bounded. Furthermore, we consider the preplanned waypoint velocity $v_{t}\left(t\right)={\dot{p}}_{t}\left(t\right)$ to be known. The leader HOV can acquire the waypoint relative position $e_{t}=p_{t}-p_{n}$ and broadcast relative position information to all follower escort AUVs.

## 3. Formation Obstacle Avoidance

Since the escort target is in motion, the cooperative escort problem for a moving underwater target by multiple AUVs can be framed as a formation tracking problem. Tracking of the moving target can be achieved by constructing a suitable objective function. Additionally, the underwater environment contains various obstacles, such as rocks, seamounts, and large marine organisms. This requires submersibles to perform cooperative obstacle avoidance during the mission. This obstacle avoidance capability can be implemented using the APF algorithm, however, traditional APF methods have inherent limitations. Common defects include the Goal Non-reachable With Obstacle Nearby (GNWON) problem [47], which arises when the goal is too close to an obstacle or the obstacle has an excessively large influence distance. In such cases, the repulsive force near the goal exceeds the attractive force towards the goal, preventing the vehicle from reaching it. Another issue arises when the goal is too far from the initial position, the attractive force near the initial point becomes excessively strong, leading to a high collision risk when obstacles are present. This study proposes a novel potential field function incorporating an adaptive mechanism by analysing the theoretical definition of the potential field function in traditional APF algorithms. This enhanced function will be used in escort formation trajectory planning.

### 3.1. Modified Attractive Potential Field

The planned trajectory waypoint is recorded as $p_{t}$. During the formation movement, the core control logic is as follows: the moving target tracks the planned trajectory waypoint $p_{t}$, while the escort AUVs track the moving target. This hierarchical tracking mechanism enables the entire formation to achieve coordinated movement and obstacle avoidance. In the proposed improved adaptive APF approach, the modified attractive potential field function is defined as follows [48]

(20) $$U_{att}\left(p_{t}\right)=\left\{\begin{array}{cc} \frac{1}{2}k_{att}d^{2}\left(p_{t},p_{g}\right), & d\left(p_{t},p_{g}\right)\leq \rho_{g}\\ k_{att}\rho_{g}d\left(p_{t},p_{g}\right)-\frac{1}{2}k_{att}\rho_{g}^{2}, & d\left(p_{t},p_{g}\right)>\rho_{g} \end{array}\right.$$

Correspondingly, the modified attractive force function is given by

(21) $$\begin{array}{c} F_{att}\left(p_{t}\right)=-\nabla U_{att}\left(p_{t}\right)=\left\{\begin{array}{cc} -k_{att}d\left(p_{t},p_{g}\right)\frac{\partial d(p_{t},p_{g})}{\partial p_{t}}, & d\left(p_{t},p_{g}\right)\leq d_{g}\\ -k_{att}d_{g}\frac{\partial d(p_{t},p_{g})}{\partial p_{t}}, & d\left(p_{t},p_{g}\right)>d_{g} \end{array}\right. \end{array}$$

where $\rho_{g}$ denotes the boundary distance parameter of the modified attractive potential field. When the distance value $d(p_{t},p_{g})$ between the waypoint $p_{t}$ and the goal $p_{g}$ exceeds $\rho_{g}$, Equations (20) and (21) indicate two key characteristics. First, the growth rate of the attractive potential field slows down. Second, the attractive force remains constant. This design addresses the issue of excessively large attractive potential values when the waypoint (or formation system) is far from the goal. Furthermore, if obstacles exist near the initial position of the waypoint, the modified attractive potential field generates a smaller potential value than the traditional APF does, while the magnitude of the attractive force remains unchanged. This balance between reduced potential and constant force enables the waypoint trajectory to smoothly avoid nearby obstacles.

### 3.2. Modified Repulsive Potential Field

To address the GNWON issue, a modified repulsion potential field has been proposed in [49]. This improvement incorporates two key modifications: first, the relative distance between the waypoint $p_{t}$ and the goal $p_{g}$ is introduced, and second, an adaptive factor *h* (h>0) is added. Consequently, the modified repulsive potential field is now correlated with both the distance between the waypoint and the obstacle, and the distance between the waypoint and the goal. The function of the modified adaptive repulsive potential field is given by

(22) $$\begin{array}{c} U_{rep}\left(p_{t}\right)=\left\{\begin{array}{cc} \frac{1}{2}k_{rep}{\left(\frac{1}{d(p_{t},p_{obs})}-\frac{1}{\rho_{0}}\right)}^{2}d^{h}\left(p_{t},p_{g}\right), & 0\leq d\left(p_{t},p_{obs}\right)\leq \rho_{0}\\ 0, & d\left(p_{t},p_{obs}\right)>\rho_{0} \end{array}\right. \end{array}$$

Correspondingly, the modified repulsion force is expressed as

(23) $$\begin{array}{c} F_{rep}\left(p_{t}\right)=-\nabla U_{rep}\left(p_{t}\right)=\left\{\begin{array}{cc} F_{rep1}\left(p_{t}\right)+F_{rep2}\left(p_{t}\right), & 0\leq d\left(p_{t},p_{obs}\right)\leq \rho_{0}\\ 0, & d\left(p_{t},p_{obs}\right)>\rho_{0} \end{array}\right. \end{array}$$

When the target escort formation system operates within the effective range of the repulsive force, the modified repulsive force consists of two distinct components, as detailed below.

(24) $$F_{rep1}\left(p_{t}\right)=k_{rep}\left(\frac{1}{d(p_{t},p_{obs})}-\frac{1}{\rho_{0}}\right)\frac{d^{h}\left(p_{t},p_{g}\right)}{d^{2}\left(p_{t},p_{obs}\right)}\cdot \frac{\partial d(p_{t},p_{obs})}{\partial (p_{t})}$$

(25) $$\begin{array}{c} F_{rep2}\left(p_{t}\right)=-\frac{1}{2}k_{rep}{\left(\frac{1}{d(p_{t},p_{obs})}-\frac{1}{\rho_{0}}\right)}^{2}\cdot h\cdot d^{h-1}\left(p_{t},p_{g}\right)\cdot \frac{\partial d(p_{t},p_{g})}{\partial (p_{t})} \end{array}$$

**Figure 3:**
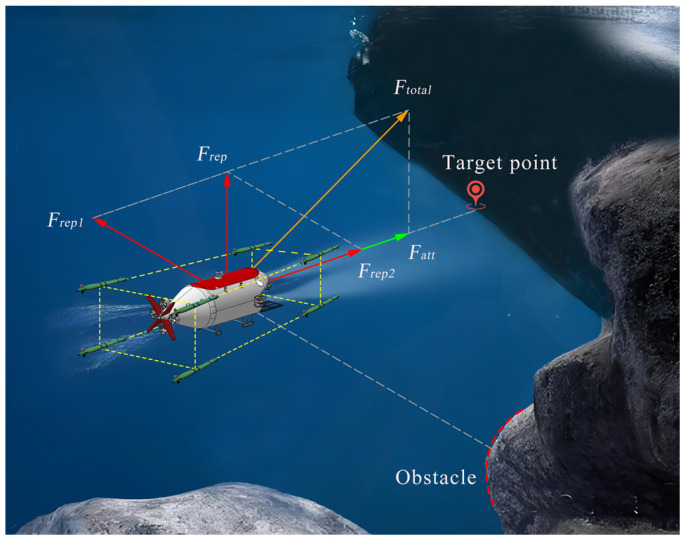
The force analysis of the target escort formation system in the modified force field.

The two vector components $F_{rep1}$ and $F_{rep2}$ of the modified repulsive force have different directions: the former points from the obstacle towards the waypoint and the latter from the waypoint towards the goal. As illustrated in Figure 3, where $F_{total}$ denotes the resultant force of the modified potential field.

The modified repulsive function decreases as the relative distance $d(p_{t},p_{g})$ between the waypoint and the goal increases, and the rate of this decrease is affected by the value of the adaptive factor *h*. Three key scenarios are analyzed below to verify that the GNWON problem has been resolved.

When 0<h<1 and the target escort formation system is close to the goal, i.e., $d(p_{t},p_{g})\to 0$, the first repulsive component $F_{rep1}\left(p_{t}\right)\to 0$ decreases as the formation moves away from the obstacle or the obstacle’s influence diminishes, while the second repulsive component $F_{rep2}\left(p_{t}\right)\to \infty$ increases. The resultant force $F_{total}\left(p_{t}\right)$ remains positive and points from the formation to the goal, ensuring the formation can reach the goal point smoothly.

When h=1 and $d(p_{t},p_{g})\to 0$. The first repulsive component approaches zero ($F_{rep1}\left(p_{t}\right)\to 0$), and the second repulsive component approaches a constant value ($F_{rep2}\left(p_{t}\right)\to \delta$). The resultant force $F_{total}\left(p_{t}\right)$ also remains positive and directed towards the goal, enabling the formation to smoothly reach the goal point.

When h>1 and $d(p_{t},p_{g})\to 0$. The modified repulsive function $F_{rep}\left(p_{t}\right)$ also decreases gradually and eventually $F_{rep}\left(p_{t}\right)\to 0$. Under the combined effect of the attractive potential field, the formation system moves along the resultant potential field and reaches the goal smoothly.

### 3.3. Adaptive Scaling Factor Adjustment

The selection of scaling factors is crucial to the collision-free target escort formation maneuver. Improperly chosen scaling factors may result in failure to reach the goal or avoid collisions. To address this issue, a proposed adaptive scaling factor approach ensures that the algorithm can perform the required tasks (reaching the goal and avoiding collisions) within a defined distance of obstacles while accounting for both the environment and the physical dimensions of the target escort formation.

The scaling factor $k_{att}$ for the attractive potential in (11) is calculated based on an assumed maximum deceleration $a_{max}$, under the premise that there are no obstacles in the vicinity of the formation. Its expression is given by

(26) $$k_{att}=\frac{\sqrt{2a_{\max}\rho_{g}}}{\rho_{g}}$$

where $\rho_{g}$ was defined in (20). Selecting the repulsive potential scaling factor $k_{rep}$ in (13) is more complex. In this study, the Widrow–Hoff rule, which was developed based on the gradient descent method [50], is adopted to derive the adaptation equation for $k_{rep}$. The Widrow–Hoff rule was originally designed for training neural networks with linear activation functions. Its core objective is to minimize the mean squared error (MSE) *E* between the desired output $y_{d}$ and the actual output $y_{out}$, where $y_{out}$ is determined by the current input $x_{j}$ and input weight $w_{j}$. The MSE is defined as

(27) $$E\left(w\right)=\frac{1}{2}{\tilde{y}}^{2}=\frac{1}{2}{\left(y_{d}-y_{out}\right)}^{2}=\frac{1}{2}{\left(y_{d}-\sum_{j}x_{j}w_{j}\right)}^{2}$$

The weight update rule for minimizing *E* is given by

(28) $$w_{j}\left(k+1\right)=w_{j}\left(k\right)-\mu \tilde{y}\left(k\right)x_{j}\left(k\right)$$

where $w_{j}\left(k+1\right)$ and $w_{j}\left(k\right)$ are the weights of *j*th input at the k+1th and *k*th iterations, respectively. μ denotes the adaptation gain (which controls the convergence speed of the weight updates), and $\tilde{y}\left(k\right)=y_{d}-y_{out}\left(k\right)$ is the error at the *k*th iteration [51]. To adaptively adjust $k_{rep}$, the Widrow–Hoff rule is modified and implemented online. The adaptation formula for $k_{rep}$ is

(29) $$k_{rep}\left(k+1\right)=k_{rep}\left(k\right)+\gamma \cdot \left(d_{safe}-d_{\min}\left(k\right)\right)$$

with

$$d_{\min}\left(k\right)=\min_{i}∥p_{t}\left(k\right)-p_{obs}^{i}\left(k\right)∥$$

where *k* denotes the algorithm iteration index, $d_{\min}\left(k\right)$ is the minimum distance from the waypoint to any obstacle at iteration *k* (with $p_{t}\left(k\right)$ representing the waypoint’s position, and $p_{obs}^{i}\left(k\right)$ the position of the *i*th obstacle), and γ>0 is the adaptation gain for $k_{rep}$. It is important to note that the value of $k_{rep}$ calculated via (29) must be bounded within upper and lower limits to avoid unbounded growth or decay from continuous integration. An insufficient $k_{rep}$ cannot guarantee collision avoidance, while an excessively large $k_{rep}$ may prevent the formation from reaching the goal. To determine these boundaries, we build on the analytical calculation method for $k_{rep}$ proposed in [40], with constraints derived from the following two key requirements.

1. When the distance between the current waypoint and an obstacle equals the safety margin $d_{safe}$, the algorithm should generate a repulsive force that causes the waypoint to move at the maximum reverse velocity $v_{t}^{\max,rev}$ (away from the obstacle). This gives
  (30) $$k_{rep}^{lower}=\frac{m{\left(v_{t}^{\max,rev}\right)}^{2}}{2d_{safe}^{2}}$$
2. When the current waypoint’s distance to an obstacle equals $\rho_{0}-\Delta \rho$ (where $\rho_{0}$ was given in (13), which denotes the obstacle reaction margin, and Δρ is a small buffer distance), the algorithm must ensure that the waypoint can still move towards the goal at the maximum forward velocity $v_{t}^{\max,fwd}$. This gives
  (31) $$k_{rep}^{upper}=\frac{m[{\left(v_{t}^{\max,fwd}\right)}^{2}-{\left(v_{t}^{\max,rev}\right)}^{2}]}{2{\left(\rho_{0}-\Delta \rho \right)}^{2}}$$
  with
  $$\Delta \rho \ll \rho_{0}$$
  where *m* represents the mass of the submersible. Defining upper and lower limits for $k_{rep}$ enables the algorithm to stop $k_{rep}$ adaptation in two scenarios: (a) when there are no obstacles around the formation (rendering repulsive force unnecessary), and (b) when the formation is in narrow corridors with nearby obstacles (preventing excessive repulsive force from trapping the formation).

## 4. Cooperative Escorting Formation Control Scheme

This section proposes a distance-based formation maneuvering strategy for multi-submersible systems, employing a backstepping controller employed as the auxiliary controller module. The design and analysis process is structured as follows: first, an auxiliary controller based on rigid graph theory is developed using the backstepping technique; second, the feasibility and stability of the proposed formation controller are analyzed via Lyapunov stability theory.

### 4.1. Design of the Formation Controller

We consider the target escort formation control problem of *n* submersibles operating in a 3D space. First, we first rewrite the Euler–Lagrange dynamic model (9) as follows:

(32a) $${\dot{p}}_{i}=v_{i}$$

(32b) $$M_{i}\left(p_{i}\right){\dot{v}}_{i}=\tau_{i}-C_{i}\left(p_{i},v_{i}\right)v_{i}-D_{i}\left(p_{i}\right)v_{i}$$

where $v_{i}\in R^{3}$ denotes the translational velocity (hand velocity) of the *i*th submersible relative to the Earth-fixed frame.

For any two connected submersibles i,j∈V, their relative position is defined as

(33) $${\tilde{p}}_{ij}=p_{i}-p_{i},\;\;\;\left(i,j\right)\in E^{*}$$

and let $\tilde{p}=\left(\ldots ,{\tilde{p}}_{ij},\ldots \right)\in R^{3l}$ where the ordering of $\tilde{p}$ terms is consistent with the edge function defined in (1). The relative position error is then expressed as

(34) $${\tilde{p}}_{ij}=p_{i}-p_{i},\;\;\;\left(i,j\right)\in E^{*}$$

where $d_{ij}\left(t\right)$ was defined in (15) is the time-varying desired relative position corresponding to edge (i,j). Combining Equations (34) and (32a), we can derive the dynamics of the distance error.

(35) $${\dot{e}}_{ij}=\frac{d}{dt}\left(\sqrt{{\tilde{p}}_{ij}^{T}{\tilde{p}}_{ij}}\right)-{\dot{d}}_{ij}=\frac{{\tilde{p}}_{ij}^{T}\left(v_{i}-v_{j}\right)}{e_{ij}+d_{ij}}-{\dot{d}}_{ij}$$

We introduce a Lyapunov candidate function referenced in [42,52]

(36) $$W_{ij}=\frac{1}{4}\beta_{ij}^{2}$$

where

(37) $$\beta_{ij}={∥{\tilde{p}}_{ij}∥}^{2}-d_{ij}^{2},\left(i,j\right)\in E^{*}$$

Using (34), (37) can be rewritten as

(38) $$\beta_{ij}=e_{ij}\left(∥{\tilde{p}}_{ij}∥+d_{ij}\right)=e_{ij}\left(e_{ij}+2d_{ij}\right)$$

Since $∥{\tilde{p}}_{ij}∥\geq 0$ (or equivalently, $e_{ij}\geq -d_{ij}$), it is straightforward to verify that $\beta_{ij}=0$ if and only if $e_{ij}=0$. Thus, (36) is positive definite and radially unbounded with respect to $e_{ij}$.

Next, we define the following Lyapunov candidate function

(39) $$W\left(e\right)=\sum_{\left(i,j\right)\in E^{*}}W_{ij}\left(e_{ij}\right)$$

where $e=\left(\ldots ,e_{ij},\ldots \right)\in R^{l}$, the ordering of it terms is consistent with (1). Taking the time derivative of (39) along the trajectory of (35), we obtain

(40) $$\dot{W}=\sum_{\left(i,j\right)\in E^{*}}e_{ij}\left(e_{ij}+2e_{ij}\right)[{\tilde{p}}_{ij}^{T}\left(v_{i}-v_{j}\right)-d_{ij}{\dot{d}}_{ij}]$$

Using (1), (2), and (34), that (40) can be simplified to

(41) $$\dot{W}=\beta^{T}\left(R\left(p\right)v-d_{v}\right)$$

where $v=\left(v_{1},\ldots ,v_{n}\right)\in R^{3n}$, $\beta =\left(\ldots ,\beta_{ij},\ldots \right)\in R^{l}$, and $d_{v}=\left(\ldots ,d_{ij}{\dot{d}}_{ij},\ldots \right)\in R^{l},\left(i,j\right)\in E^{*}$. The terms in β and $d_{v}$ follow the same ordering as (1).

Following the backstepping technique, we introduce an intermediate virtual control variable

(42) $$\tilde{v}=v-v_{f}$$

where $v_{f}\in R^{3n}$ denotes the fictitious hand velocity input. denotes the fictitious hand velocity input. We further define a Lyapunov function to account for the velocity error and the parameter estimation errors.

(43) $$W_{m}\left(e,\tilde{v},\tilde{\phi }\right)=W\left(e\right)+\frac{1}{2}{\tilde{v}}^{T}M\left(p\right)\tilde{v}+\frac{1}{2}{\tilde{\phi }}^{T}\Gamma^{-1}\tilde{\phi }$$

where $M\left(p\right)=\mathrm{diag}(M_{1}\left(p_{1}\right),\ldots ,M_{n}\left(p_{n}\right))$, $\Gamma \in R^{12n\times 12n}$ is constant, diagonal, and positive definite. Taking the time derivative of (43), we get

(44) $$\begin{array}{cc} {\dot{W}}_{m} & =\dot{W}\left(e\right)+\frac{1}{2}{\tilde{v}}^{T}\dot{M}\left(p\right)\tilde{v}+{\tilde{v}}^{T}M\left(p\right)\dot{\tilde{v}}+{\tilde{\phi }}^{T}\Gamma^{-1}\dot{\tilde{\phi }}\\ \; & =\beta^{T}\left(R\left(p\right)v-d_{v}\right)+\frac{1}{2}{\tilde{v}}^{T}\dot{M}\left(p\right)\tilde{v}+{\tilde{v}}^{T}M\left(p\right)\left(\dot{v}-{\dot{v}}_{f}\right)+{\tilde{\phi }}^{T}\Gamma^{-1}\left(\dot{\hat{\phi }}-\dot{\phi }\right)\\ \; & =\beta^{T}\left(R\left(p\right)v-d_{v}\right)+\frac{1}{2}{\tilde{v}}^{T}\dot{M}\left(p\right)\tilde{v}+{\tilde{v}}^{T}\left[\tau -C\left(p,\dot{p}\right)\dot{p}-D\left(p\right)\dot{p}-M\left(p\right){\dot{v}}_{f}\right]\\ \; & +{\tilde{\phi }}^{T}\Gamma^{-1}\left(\dot{\hat{\phi }}-\dot{\phi }\right)\\ \; & =\beta^{T}\left(R\left(p\right)v-d_{v}\right)+\frac{1}{2}{\tilde{v}}^{T}\dot{M}\left(p\right)\tilde{v}-C\left(p,\dot{p}\right)\tilde{v}+{\tilde{v}}^{T}\left[\tau -C\left(p,\dot{p}\right)v_{f}+D\left(p\right)\dot{p}-M\left(p\right){\dot{v}}_{f}\right]\\ \; & +{\tilde{\phi }}^{T}\Gamma^{-1}\left(\dot{\hat{\phi }}-\dot{\phi }\right) \end{array}$$

Using Property 2 (skew-symmetry of ${\tilde{v}}^{T}\left(\dot{M}\left(p\right)-2C\left(p,\dot{p}\right)\right)\tilde{v}=0$, ) and Property 3 (linear parameterization) of Euler–Lagrange systems, and assuming the parametric uncertainty term $\dot{\phi }=0$, then (44) can be transformed into

(45) $$\begin{array}{cc} {\dot{W}}_{m} & =\beta^{T}\left(R\left(p\right)v_{f}-d_{v}\right)+{\tilde{v}}^{T}\left[\tau -Y\left(p,\dot{p},v_{f},{\dot{v}}_{f}\right)\left(\hat{\phi }-\tilde{\phi }\right)+R^{T}\left(p\right)\beta \right]\\ \; & +{\tilde{\phi }}^{T}\Gamma^{-1}\left(\dot{\hat{\phi }}-\dot{\phi }\right)\\ \; & =\beta^{T}\left(R\left(p\right)v_{f}-d_{v}\right)+{\tilde{v}}^{T}\left[\tau -Y\left(p,\dot{p},v_{f},{\dot{v}}_{f}\right)\hat{\phi }+R^{T}\left(p\right)\beta \right]\\ \; & +{\tilde{\phi }}^{T}\Gamma^{-1}\dot{\hat{\phi }}+\tilde{\phi }Y^{T}\left(p,\dot{p},v_{f},{\dot{v}}_{f}\right)\tilde{v} \end{array}$$

The following theorem presents the control law for the multi-AUV cooperative escort formation maneuvering problem.

**Theorem** **1.** *Consider the multi-AUV cooperative target escort formation framework $F\left(t\right)=(G^{*},p\left(t\right))$, and let ε represents a sufficiently small positive constant, and $\mathrm{dist}(p,I\left(p^{*},E_{g}\right))\leq \varepsilon$ for all p, where additional details about the compact set $I(p^{*},E_{g})$, can be found in [53]. Assume the initial conditions of (32) satisfies $\tilde{p}\left(0\right)\in S$, where*

$$S=\{\tilde{p}\in R^{3l}|W\left(e\right)\leq c\}$$

*and c is a sufficiently small positive constant dependent on the value of ε. Then, selecting the control input as*

(46a) $$\tau =-k_{a}\tilde{v}+Y\left(p,\dot{p},v_{f},{\dot{v}}_{f}\right)\hat{\phi }-R^{T}\left(p\right)\beta$$

(46b) $$v_{f}=R^{†}\left(p\right)\left(-k_{v}\beta +d_{v}\right)+\left(1_{n}⊗v_{d}\right)$$

(46c) $$v_{d}=v_{t}+k_{t}e_{t}$$

(46d) $$\dot{\hat{\phi }}=-\Gamma Y^{T}\left(p,\dot{p},v_{f},{\dot{v}}_{f}\right)\tilde{v}$$

*in (46), τ is defined in (9), $k_{a}$ is a positive constant, as are $k_{v}$ and $k_{t}$, and $R^{†}\left(p\right)=R^{T}\left(p\right){\left[R\left(p\right)R{\left(p\right)}^{T}\right]}^{-1}$ represents the Moore-Penrose Pseudoinverse of R(p), $\hat{\phi }=\left({\hat{\phi }}_{1},{\hat{\phi }}_{2},\ldots {\hat{\phi }}_{n}\right)$. Here, $v_{t}={\dot{p}}_{t}\left(t\right)$ represents the velocity of pre-planned trajectory waypoint, and $e_{t}=p_{t}-p_{n}$ denotes the trajectory tracking error of the leader HOV. By selecting the control gains and design parameters appropriately, the distance error e, the auxiliary variable $\tilde{v}$, and the parameter estimation error $\tilde{\phi }$ can be made uniformly ultimately bounded (UUB). This ensures that the equilibrium point of the closed-loop system is exponentially stable, thereby guaranteeing satisfaction of the control objectives in Equations (16) and (17).*

### 4.2. Stability Analysis

Without loss of generality, the stability of the proposed controller is verified using Lyapunov stability theory. Substituting (46a–c) into (45) yields

(47) $$\begin{array}{c} {\dot{W}}_{m}=-k_{v}\beta^{T}\beta -k_{a}{\tilde{v}}^{T}\tilde{v}=-4k_{v}W\left(e\right)-k_{a}{\tilde{v}}^{T}\tilde{v}\\ \leq -k_{v}W\left(e\right)-k_{a}{∥\tilde{v}∥}^{2} \end{array}$$

Since W(e) is a Lyapunov candidate function, it is clear that $-4k_{v}W\left(e\right)$ is negative definite, and that ${∥\tilde{v}∥}^{2}$ is the square of the Euclidean norm, the sign of the term is positive. From the above expressions, it can be clearly seen that $k_{a}$, $k_{v}$ are positive constants, and that, from (47), ${\dot{W}}_{m}$ is negative definite. For all cases where t≥0, ${\dot{W}}_{m}\leq 0$, so for t≥0, $W_{m}$ remains the same or decreases. Note that $W_{m}$ has finite limits as *t* tends to infinity.

From Equations (43) and (47), we have $W_{m}\geq 0$ and ${\dot{W}}_{m}\leq 0$. It follows that, for t≥0, there exists functions β(t), $\tilde{v}\left(t\right)$, $\tilde{\phi }\left(t\right)\in L_{\infty }$, where $L_{\infty }$ represents signal infinity norm. From (34) and (37), it can be seen that, as t→∞, both $\tilde{p}\left(t\right)\in L_{\infty }$ and $e\left(t\right)\in L_{\infty }$. These results and (46b) imply that $v_{f}\in L_{\infty }$, thus $v\left(t\right)\left(=\dot{p}\left(t\right)\right)\in L_{\infty }$ from (42). Using (46a) and the fact that θ and φ appear solely through trigonometric functions, it can be see that the result of $\tau \left(t\right)\in L_{\infty }$. Furthermore, from the expression of J(θ,φ), one know *J* and $J^{-1}$ are bounded, we know $e\left(t\right)\in L_{\infty }$ from (7), it also becomes feasible to know ${\bar{\tau }}_{i}\left(t\right)\in L_{\infty }$. From (9), one have that $\ddot{p}\left(t\right)\in L_{\infty }$. Then ${\dot{p}}_{ci}\left(t\right)\in L_{\infty }$ is evident from (3a) and (4). From (3b), it is obvious that ${\dot{\eta }}_{i}\left(t\right)\in L_{\infty }$. Thus, the stability proof is complete.

## 5. Simulation and Results Analysis

### 5.1. Simulation Setup and Sarameters

**Figure 4:**
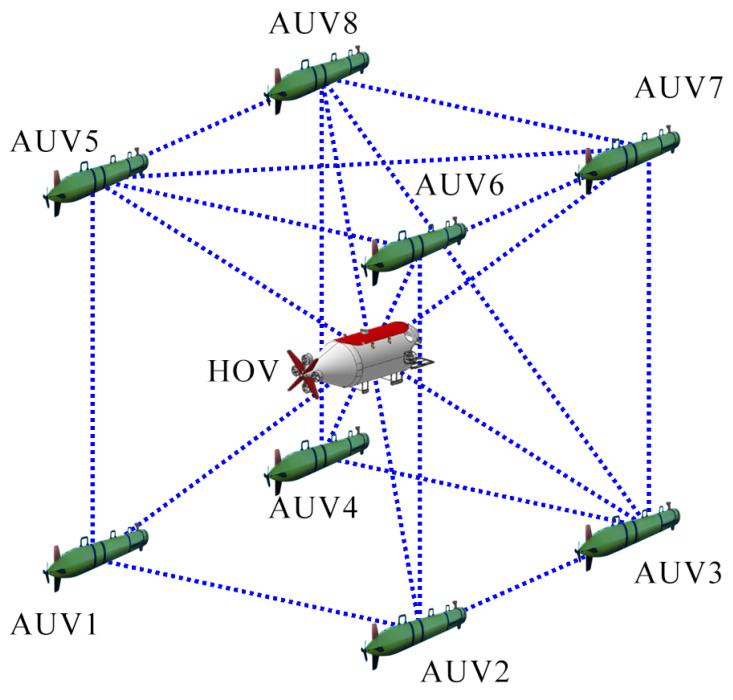
The desired target escort formation structure.

The effectiveness of the proposed cooperative formation escort algorithm is validated through numerical simulations conducted in MATLAB R2024a on a Windows 11 PC. The simulation scenario involves an undirected connected communication network, where each node represents a submersible. The desired formation framework $F^{*}=\left(G^{*},p^{*}\right)$ is a regular cubic structure (the nominal formation) consisting of eight AUVs and one HOV, as illustrated in Figure 4. AUV dynamic parameters: For all i=1,2,…,8, the parameters are set as $m_{i}=10.0\;\mathrm{kg}$, $I_{1i}=0.8\;\mathrm{kg}\cdot m^{2}$, $I_{2i}=0.03\;\mathrm{kg}\cdot m^{2}$, ${\bar{D}}_{i}=\mathrm{diag}\left\{0.5\;\mathrm{kg}/s,0.05\;\mathrm{kg}\cdot m^{2}/s,0.8\;\mathrm{kg}\cdot m^{2}/s\right\}$, and $L_{i}=0.5\;m$. The initial location of the *i*th submersible, $p_{i}\left(0\right)$, is randomly sampled near its desired initial position $p_{i}^{*}$ as, $p_{i}\left(0\right)=p^{*}+\left[\mathrm{rand}\left(0,1\right)-0.5I\right]$, where rand(0,1) produces a random 3×1 vector with elements uniformly distributed over (0,1), *I* is the 3×1 vector of ones, and 0.5 scales the perturbation range. The initial orientation $\theta_{i}\left(0\right)$ (pitch) and $\varphi_{i}\left(0\right)$ (yaw) are randomly set within [0,2π). The initial parameter estimates are set to zero, i.e., ${\hat{\phi }}_{i}\left(0\right)=0$. The initial translational velocities of all the submersibles are $v_{i}\left(0\right)=\left[0,-0.15,0.45,-0.35,0.35,0.5,-0.25,0.35,-0.25\right]$ m/s, where i=1,2,…,9. The control gains are set to $k_{a}=5$, $k_{v}=2$, $k_{t}=0.5$ and $\Gamma_{i}=10I_{i}$. Environment: A 3D underwater space with dimensions (65×65×55) m^3^.

### 5.2. Escorting Formation Spiral Descent

**Figure 5:**
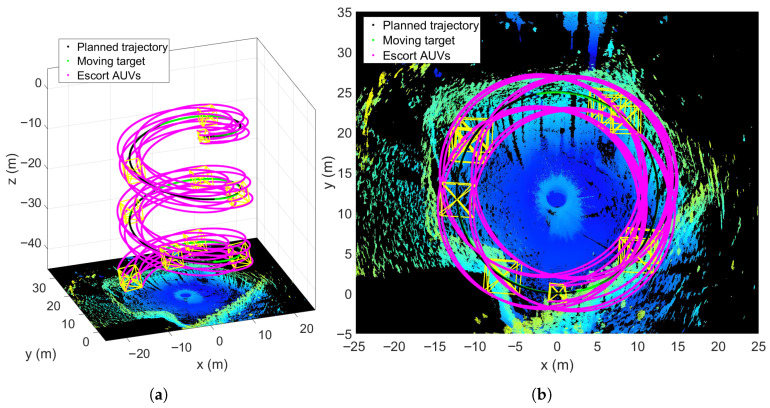
Spiral descent movement trajectory of the escort formation system. (**a**) 3D trajectories. (**b**) x−y plane trajectories.

The first simulation scenario involves a multi-AUV formation escorting the target (HOV) in a spiral descent motion to guide it to an unobstructed underwater area for detection. The simulation runs for 200 s, during which the cooperative escort performance is evaluated in full. Figure 5 visualizes the motion states of all the AUVs and the HOV, where the Figure 5a shows the 3D trajectories, while Figure 5b presents the 2D projections on the x−y plane. In Figure 5, the black dotted line indicates the trajectory of planned waypoint trajectory, and the green dotted line denotes the moving target’s actual trajectory, and the pink dotted lines represent the escort AUVs’ trajectories. As can be seen in Figure 5, the moving target can accurately tracks the spiral reference trajectory. All escort AUVs maintain the desired formation while tracking the moving target, ensuring it is continuously surrounded, which is a key indicator of successful escorting.

**Figure 6:**
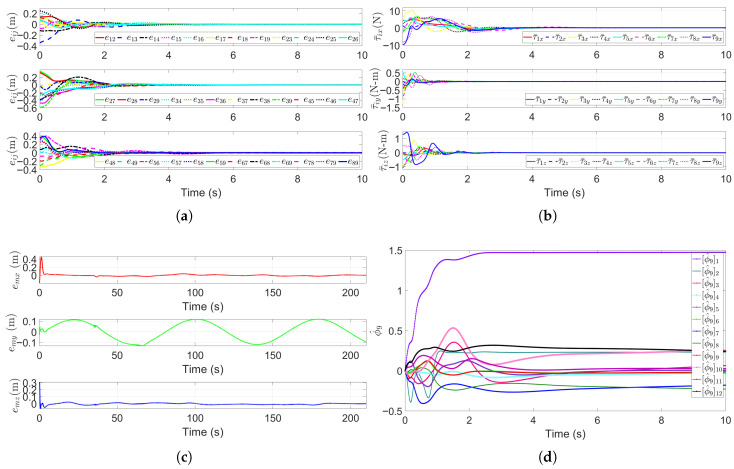
Performance metrics of the escort formation system. (**a**) Relative distance errors $e_{ij}\left(t\right),i,j\in V^{*}$ between any two submersibles. (**b**) Formation control inputs ${\bar{\tau }}_{i}$ (in *x*, *y* and *z* directions) for each submersible. (**c**) Trajectory tracking error $e_{t}$ of the leader HOV (submersible 9). (**d**) Sample of parameter estimates ${\hat{\phi }}_{9}$ for the leader HOV.

Figure 6 shows the performance metrics of the target escort formation system. Figure 6a plots the relative distance errors, $e_{ij}\left(t\right),i,j\in V^{*}$, between any two submersibles. These errors converge to near-zero within four seconds, confirming that the rigidity graph-based formation control using rigidity constraints enforces precise geometry. Figure 6b shows the control inputs of submersibles, labelled as ${\bar{\tau }}_{i}\left(t\right)$. These inputs exhibit significant initial fluctuations (to correct initial position errors, with peaks reflecting system inertia), but stabilize to near-zero values once the formation is established. This is consistent with expectations, as only minor adjustments are needed to maintain the formation’s shape. Figure 6c presents the moving target’s trajectory tracking error $e_{t}$, which remains small throughout the simulation. This demonstrates the algorithm’s ability to adapt to the time-varying spiral trajectory. Finally, Figure 6d shows the parameter estimates ${\hat{\phi }}_{9}$ for the leader moving target (labelled as submersible 9). These estimates gradually converge to stable values, thus verifying the robustness of the adaptive law (34d) against system uncertainties through asymptotic convergence.

### 5.3. Escort Formation Maneuvers in a Simple Obstacle Environments

**Figure 7:**
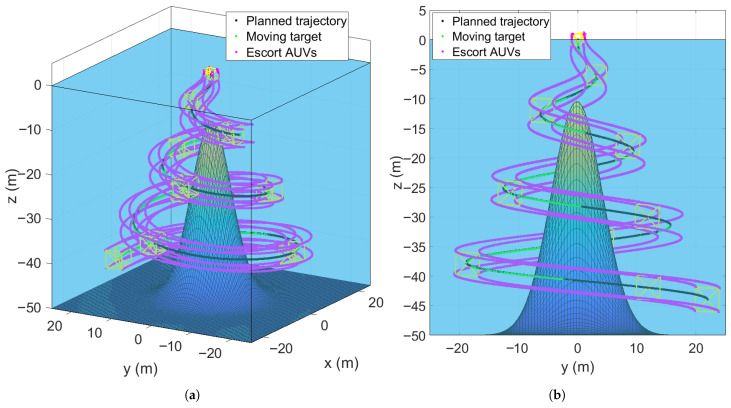
Spiral descent trajectories of the escort formation system in a single-obstacle environment. (**a**) Three-Dimensional trajectories. (**b**) y−z plane trajectories.

**Figure 8:**
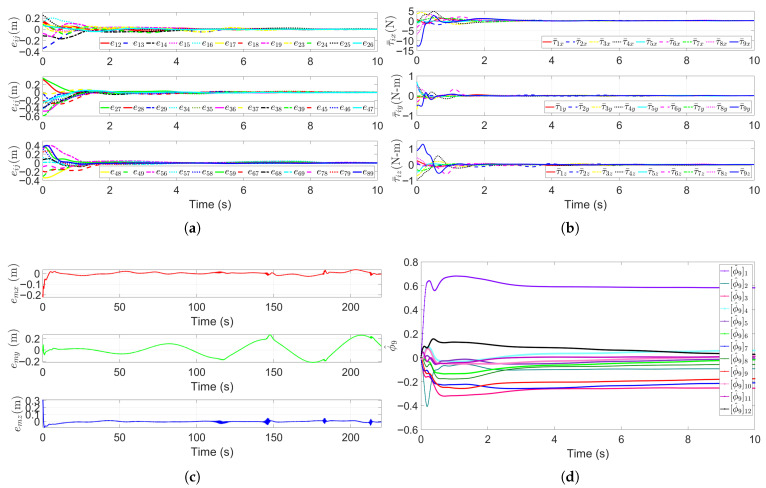
Performance metrics of the escort formation system in a single-obstacle environment. (**a**) Relative distance errors $e_{ij}\left(t\right),i,j\in V^{*}$. (**b**) Formation control inputs ${\bar{\tau }}_{i}$. (**c**) Trajectory tracking error $e_{t}$. (**d**) Sample of parameter estimates ${\hat{\phi }}_{9}$ for the leader HOV.

The second simulation scenario considers a multi-submersible formation performing a spiral descent while conducting a detection mission around a single obstacle (seamount). The maneuver control law proposed in Equation (46a) to (46d) is adopted, and the simulation results are presented in Figure 7 and Figure 8. Figure 7 illustrates the formation’s trajectory during an obstacle-aware spiral descent. Figure 7a shows the 3D trajectories of the HOV (target) and the escort AUVs, clearly demonstrating how the formation avoids the seamount. Figure 7b presents the 2D projections on the y−z plane, further demonstrating how the formation adjusts its path to bypass the obstacle while maintaining the spiral descent.

Figure 8a plots the relative distance errors $e_{ij}\left(t\right),i,j\in V^{*}$ between all connected submersibles. These errors satisfy the prescribed performance constraints and converge asymptotically to near zero over time. Initially, the errors are relatively large, this is due to the escort AUVs needing time to adjust their velocity and heading to match the HOV’s obstacle avoidance motion. As the simulation progresses, the errors decrease rapidly and, within five seconds, all distance errors stabilize near zero, confirming the formation’s ability to maintain geometric constraints during obstacle maneuvers. Figure 8b shows the formation control inputs ${\bar{\tau }}_{i}$ for each submersible in the *x*-, *y*-, and *z*-directions. These inputs initially fluctuate moderately (to compensate for initial errors and adapt to the HOV’s obstacle avoidance trajectory), but stabilize at small magnitudes once the formation reaches a steady state. This is consistent with the requirement for minimal adjustments during stable formation maintenance. Figure 8c presents the HOV’s trajectory tracking error $e_{t}$. Despite the presence of the obstacle, the tracking error remains small and bounded, verifying that the control law balances obstacle avoidance and trajectory following. Figure 8d shows the parameter estimates ${\hat{\phi }}_{9}$ for the leader HOV. The estimates gradually converge to stable values, even during the obstacle avoidance maneuver, demonstrating the robustness of the adaptive law (46a–d) against transient disturbances from obstacle interactions.

### 5.4. Escorting Formation Maneuvering in a Static Multi-Obstacle Environment

**Figure 9:**
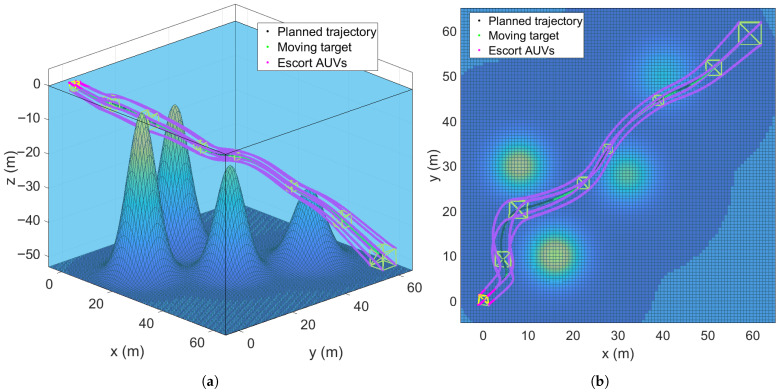
Trajectories of the escort formation system in a static multi-obstacle environment. (**a**) Three-dimensional trajectories. (**b**) x−y plane trajectories.

The underwater environment is inherently complex, with unavoidable static obstacles such as seamounts and coral reefs. To validate the robustness of algorithm in realistic scenarios, multiple static obstacles are placed in the simulation environment. The formation control and path planning method (the RG-SAPF method) proposed in this study is adopted. Additionally, we considered the scaling reconfiguration of the formation to adapt to the changes in the multi-obstacle environment. The obstacle avoidance trajectories of the escort AUV formation are visualized in Figure 9. Figure 9a shows the 3D trajectories of all submersibles, while Figure 9b presents their 2D projections on the x−y plane. As can be seen, the AUV formation rapidly converges to the desired regular cubic structure under the guidance of the RG-SAPF method. Crucially, all submersibles successfully bypass the multiple static obstacles without colliding, while maintaining the overall formation integrity and accurately tracking the pre-planned spiral descent path. Incorporating the Widrow–Hoff rule into the adaptive adjustment of the repulsive potential field scaling factor enables the algorithm to effectively avoid local minima (a common defect of traditional APF methods) and quickly identify a safe obstacle avoidance path for the formation, significantly enhancing the reliability and efficiency of obstacle avoidance.

**Figure 10:**
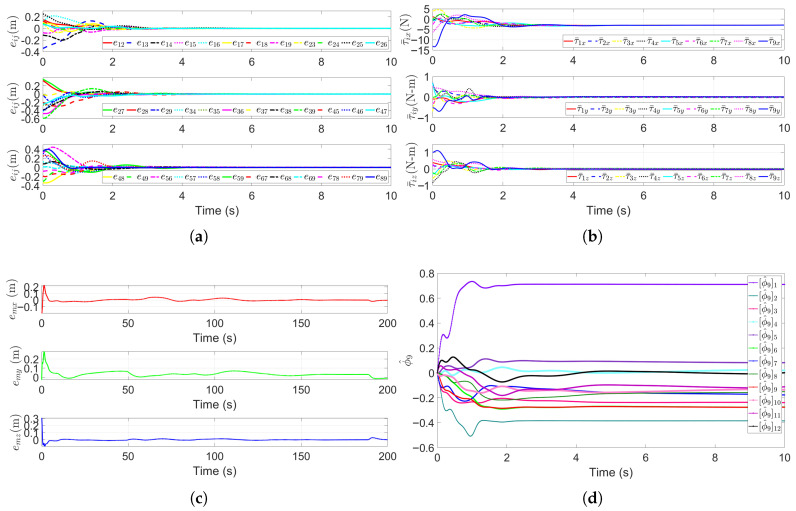
Performance metrics of the escort formation system in a static multi-obstacle environment. (**a**) Relative distance errors $e_{ij}\left(t\right),i,j\in V^{*}$. (**b**) Formation control inputs ${\bar{\tau }}_{i}$. (**c**) Trajectory tracking error $e_{t}$. (**d**) Sample of parameter estimates ${\hat{\phi }}_{9}$ for the leader HOV.

The key performance metrics of the formation system are presented in Figure 10. Figure 10a plots the relative distance errors $e_{ij}\left(t\right),i,j\in V^{*}$ between all connected submersibles. Despite the presence of multiple obstacles, these errors converge asymptotically to near-zero values, which confirms that the RG-SAPF method enforces the desired formation’s strict geometric constraints. Figure 10b shows the control inputs ${\bar{\tau }}_{i}$ (in *x*-, *y*-, and *z*-directions) for each submersible. These inputs exhibit transient fluctuations in the initial stage to adapt to obstacle avoidance, formation convergence, and formation reconfiguration, but stabilize to small magnitudes once the formation enters a steady state. This is consistent with the requirement for energy-efficient formation maintenance. Figure 10c presents the leader HOV’s trajectory tracking error $e_{t}$. The error remains small and bounded throughout the simulation, demonstrating the algorithm’s ability to balance obstacle avoidance, formation reconfiguration, and trajectory following. Figure 10d shows a sample of parameter estimates, namely the estimate of the parameter ${\hat{\phi }}_{9}$ for the leader HOV. These estimates gradually converge to stable values, even amid multiple obstacle interactions, thus verifying the adaptive law’s robustness against complex environmental disturbances.

### 5.5. Escorting Formation Maneuvering in a Dynamic Obstacle Environment

**Figure 11:**
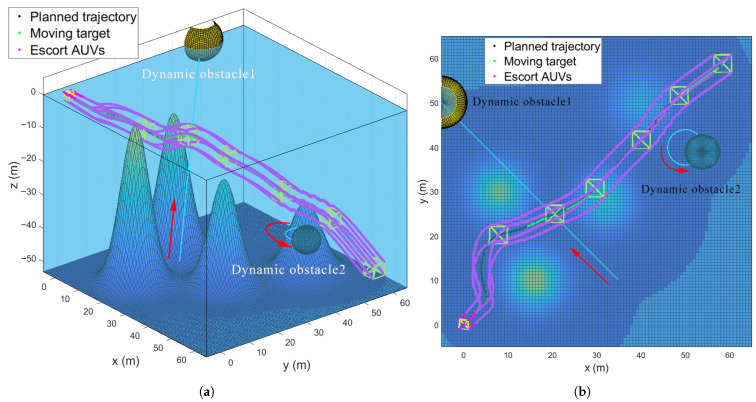
Trajectories of the escort formation system in a dynamic obstacle environment. (**a**) 3D trajectories. (**b**) x−y plane trajectories.

Dynamic obstacles (e.g., large marine organisms or floating debris) are common in real-world multi-AUV escort missions. To validate the algorithm’s capability for avoiding dynamic obstacles, this simulation builds on the static multi-obstacle environment in Section 5.4, introducing two additional dynamic obstacles. As illustrated in Figure 11, the dynamic obstacles are initialized at the positions $p_{obs1}=\left(40,10,-35\right)$, and $p_{obs2}=\left(55,40,-40\right)$, with the moving speeds of $v_{obs1}=\left(-0.1,0.2,0.2\right)$ m/s, and $v_{obs2}=\left(-\cos\left(t\right),sin\left(t\right),0\right)$ m/s, respectively. A key premise of this scenario is that the multi-AUV system uses onboard sensors to acquire real-time dynamic obstacle information (position and velocity) prior to potential encounters. The SAPF algorithm then executes dynamic obstacle avoidance with adaptive repulsive potential field adjustments. Figure 11 visualizes the formation’s trajectory during dynamic obstacle avoidance. Figure 11a shows the 3D trajectories of the HOV, the escort AUVs and the dynamic obstacles. This clearly depicts the proactive path adjustment made by the formation to avoid collisions. Figure 11b presents the 2D projections on the x−y plane, further illustrating the formation’s coordinated maneuver to bypass the moving obstacles. As can be seen, when encountering dynamic obstacles, the SAPF algorithm rapidly computes a collision-free path for the formation. Crucially, the AUVs maintain the desired regular cubic formation throughout the avoidance process while continuing to escort the HOV along the pre-planned obstacle avoidance trajectory. This fully aligns with the core objective of the cooperative escort mission.

**Figure 12:**
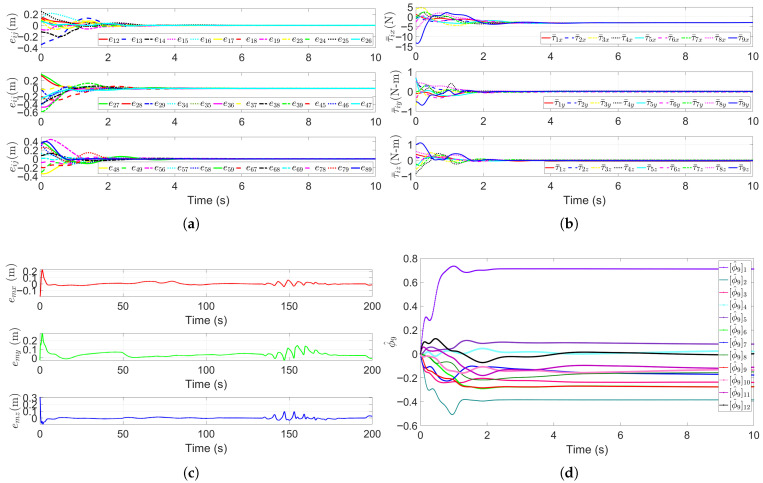
Performance metrics of the escort formation system in a dynamic obstacle environment. (**a**) Relative distance errors $e_{ij}\left(t\right),i,j\in V^{*}$. (**b**) Formation control inputs ${\bar{\tau }}_{i}$. (**c**) Trajectory tracking error $e_{t}$. (**d**) Sample of parameter estimates ${\hat{\phi }}_{9}$ for the leader HOV.

Key performance metrics for the dynamic obstacle scenario are presented in Figure 12. Figure 12a plots the relative distance errors $e_{ij}\left(t\right),i,j\in V^{*}$ between all connected submersibles. Despite interactions with dynamic obstacles, the errors converge to near-zero within four seconds and remain stable thereafter, confirming that the RG-SAPF integrated method effectively maintains geometric formation constraints during dynamic maneuvers. Figure 12b shows the control inputs ${\bar{\tau }}_{i}\left(t\right)$ for each submersible in the *x*-, *y*-, and *z*-directions. These inputs exhibit transient peaks in the initial stage to drive formation convergence and dynamic obstacle avoidance, stabilising to near-zero magnitudes once the formation enters a steady state. This reflects energy-efficient control behaviour. Figure 12c presents the HOV’s trajectory tracking error $e_{t}$. These error remains small and bounded, even during obstacle avoidance, demonstrating the algorithm’s ability to balance dynamic obstacle evasion, formation maintenance, and trajectory following. Figure 12d shows the parameter estimates, denoted by ${\hat{\phi }}_{9}$, for the leader HOV. These estimates gradually converge to stable values, remaining unaffected by dynamic obstacle disturbances. This verifies the robustness of the adaptive law in time-varying environmental conditions.

### 5.6. Comparison with the Traditional APF Algorithm

**Figure 13:**
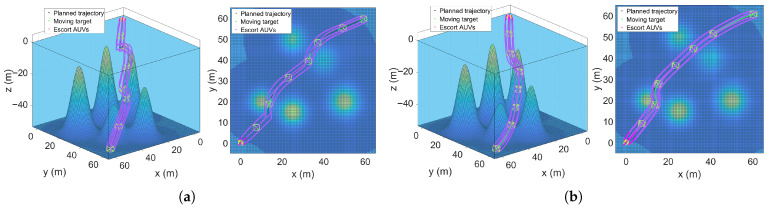
Comparison of formation escort algorithms. (**a**) RG-APF method. (**b**) RG-SAPF method.

This section demonstrates the advantages of the proposed RG-SAPF method by comparing it with the traditional APF-based formation motion planning method in a three-dimensional ocean space containing five static obstacles. Figure 13 presents the simulation results under the same simulation environment and formation motion conditions. Figure 13a shows the RG-APF algorithm simulation results and Figure 13b shows the RG-SAPF algorithm simulation results. The comparison indicators and results show that the RG-APF algorithm takes 8.47 s to run, whereas the RG-SAPF algorithm takes 8.16 s. The length of the planned obstacle avoidance path is 99.75 m and 99.04 m, respectively. The motion path length of the escorted target is 105.55 m and 103.62 m, respectively. The total path length for all the escort AUVs is 846.31 m for the RG-APF method and 823.93 m for the RG-SAPF method. Comparing indicators such as the algorithm’s running time and the trajectory path length further verifies the RG-SAPF method’s superiority in maintaining the escort formation and avoiding multiple obstacles.

## 6. Experimental  Results

To verify the feasibility of the proposed algorithm and the entire system for practical applications, a series of field experiments were conducted in an outdoor pool. Within the formation control and trajectory planning framework (RG-SAPF framework), this section validates the performance of the formation controller.

### 6.1. Experimental Platform

**Figure 14:**
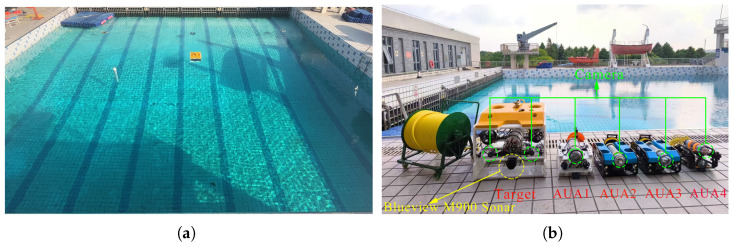
Experiment platform. (**a**) Outdoor pool test site. (**b**) AUV hardware systems.

**Figure 15:**
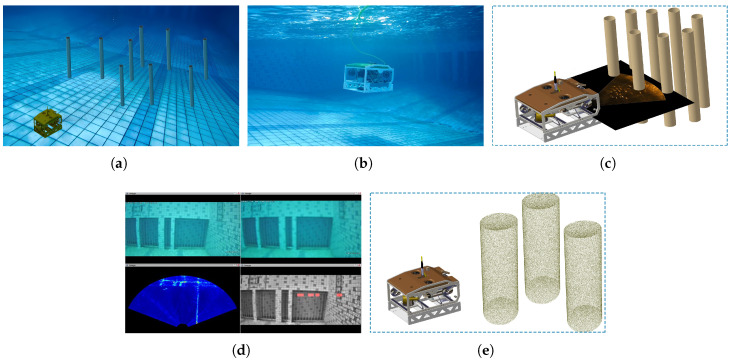
Obstacle detection and underwater environmental construction. (**a**) Target AUV (AUV 5) in the vision of an escort AUV. (**b**) Obstacle detection via sonar and cameras. (**c**) Obstacle information collection and preprocessing. (**d**) Data fusion of sonar point clouds and visual images. (**e**) 3D underwater environment modeling.

Figure 14 visualises the outdoor pool experimental system is in, where Figure 14a shows the outdoor pool test site, and Figure 14b presents the core experimental equipment. As illustrated in Figure 14b, the experimental platform’s hardware system mainly comprises the following units: (1) AUVs: Five AUVs equipped with STM32 microcontrollers and NVIDIA edge computing modules were deployed to execute the formation task. Of these, AUVs 1–4 serve as escort AUVs, while AUV 5 functions as the escorted target (HOV). Notably, AUV 5 is integrated with an Inertial Measurement Unit (IMU) to measure real-time acceleration and angular velocity, providing high-precision motion state feedback. (2) Obstacle detection module: The escorted target (AUV 5) is equipped with a Blueview M900 sonar and two underwater cameras to detect the position of obstacle, as shown in Figure 15a. Additionally, each escort AUV (AUVs 1–4) is fitted with a camera to monitor the position of the target AUV (AUV 5), as shown in Figure 15b), This ensures formation cohesion. For detailed methods of obstacle detection, environmental modelling, and sensor data processing, refer to [54]. Figure 15c–e illustrate the basic process of collecting and processing obstacle information. (3) Localization and communication unit: An centimetre-level Ultra-Wideband (UWB) positioning system is used to localise all AUVs. This system also transmits the real-time motion data (position, velocity, and orientation) of each AUV back to the onshore control center in real time, enabling closed-loop control and formation supervision [19].

### 6.2. Path Planning and Obstacle Avoidance Experiment

**Figure 16:**
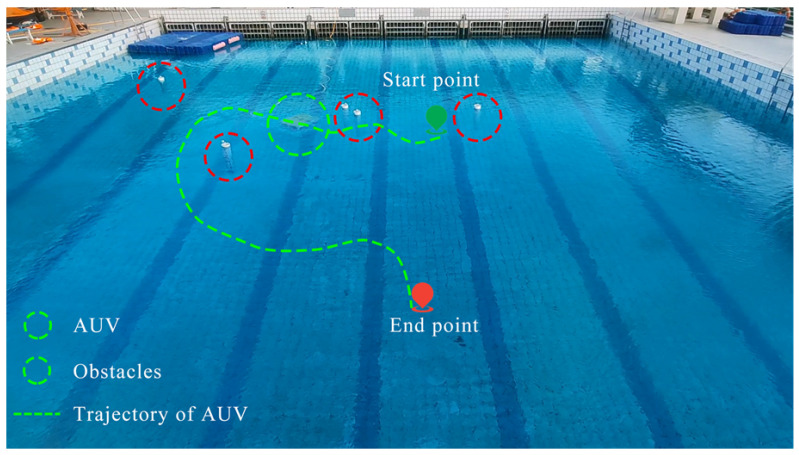
Movement trajectory of the target AUV during the maneuver and obstacle avoidance experiment.

The first experiment focuses on AUV path planning and obstacle avoidance and is conducted in an outdoor pool to validate the practical performance of the SAPF module. Figure 16 presents the movement trajectory of the target AUV (AUV 5) during the maneuver and obstacle avoidance process. As can be seen, the target AUV successfully bypasses all the preset obstacles (PVC pipes placed in the pool) without collision colliding with them, thus demonstrating the effectiveness and practicality of the proposed SAPF-based obstacle avoidance method.

**Figure 17:**
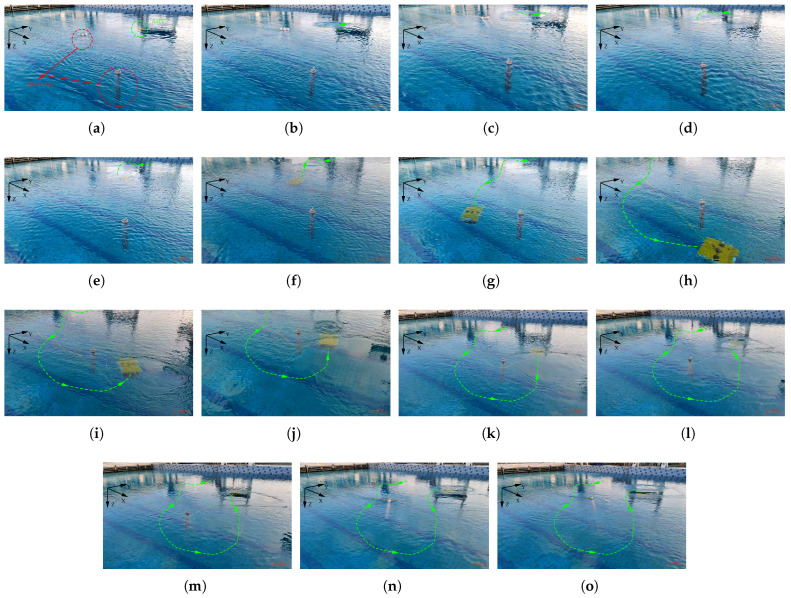
Sequential video snapshots of the target AUV’s obstacle avoidance process (captured at t=0 s to t=70 s at 5-s intervals).

To further illustrate the dynamic obstacle avoidance process, Figure 17a–o show sequential snapshots of the target AUV’s movement at discrete time intervals: (a) t=0 s; (b) t=5 s; (c) t=10 s; (d) t=15 s; (e) t=20 s; (f) t=25 s; (g) t=30 s; (h) t=35 s; (i) t=40 s; (j) t=45 s; (k) t=50 s; (l) t=55 s; (m) t=60 s; (n) t=65 s; (o) t=70 s. In these figures, the green dotted line represents the movement trajectory of the AUV, and the arrow indicates the direction of the AUV’s movement. These snapshots clearly capture the AUV’s real-time path adjustment from following the initial trajectory to detecting the obstacle and finally bypassing it without colliding, thus verifying the algorithm’s responsiveness to practical environmental disturbances.

### 6.3. Multi-AUV Formation Escort Experiment

To validate the performance of formation maintenance during escort maneuvers, a field experiment was conducted using three AUVs. The three AUVs were assigned distinct roles: one served as the escorted target (the leader AUV), while the other two acted as the escort AUVs (the followers), forming a triangular formation for the escort task. The leader AUV (the escorted target) was set to move along a straight line parallel to the positive X-axis, with a preset goal position $p_{g}=\left(25,0,0\right)$ relative to the initial origin. The two follower AUVs were required to track the leader and maintain the desired triangular formation, with predefined inter-AUV distances of $d_{12}=6$ m (distance between leader AUV 1 and follower AUV 2), $d_{13}=6$ m (distance between leader AUV 1 and follower AUV 3), and $d_{23}=10$ m (between follower AUV2 and follower AUV3). The experiment was divided into two sequential phases to verify formation convergence and maintenance:

(1) Phase 1: Formation initialization. The AUVs were first initialized at their respective starting positions. The followers were required to converge to a “guard formation” (symmetrically distributed on both sides of the leader) using two core algorithms: These were the RG-based coordination algorithm (to enforce the geometric rigidity of the triangular formation) and the leader–follower strategy (to ensure the followers tracked the leader’s state). During this phase, the leader first calculated its relative position to the goal using UWB localization data. The followers then adjusted their positions in real time based on two inputs: (a) the leader’s position and velocity information (transmitted via UWB), and (b) the predefined inter-AUV distances $d_{12}$, $d_{13}$, $d_{23}$.

(2) Phase 2: Formation escort maneuver. Once the initial triangular formation had been established, the leader started to move along the preset straight-line trajectory. The followers continued to track the leader while maintaining the desired formation shape. To enhance tracking accuracy, each follower continuously updated two key estimates using visual feedback (from onboard cameras): (a) the leader’s real-time position, and (b) the current formation configuration error (deviation from $d_{12}$, $d_{13}$, and $d_{23}$).

**Figure 18:**
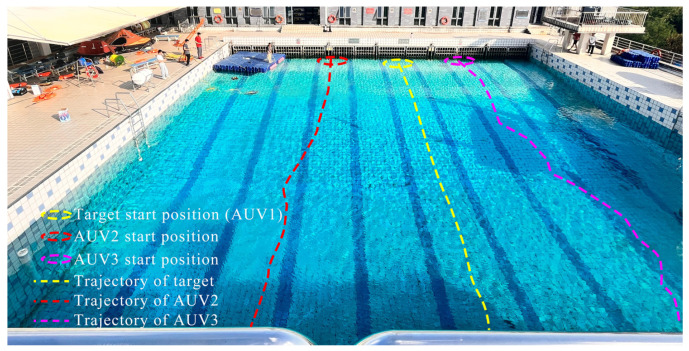
Movement trajectory of the AUVs in the formation target escort experiment.

Figure 18 presents the movement trajectories of the three AUVs under the formation controller (46). The leader AUV was observed to accurately follow the preset straight-line trajectory towards sthe goal position. The two follower AUVs were symmetrically distributed on both sides of the leader throughout the maneuver, maintaining the integrity of the triangular formation. No collisions occurred between the AUVs, confirming the safety of the RG-SAPF based coordination algorithm.

**Figure 19:**
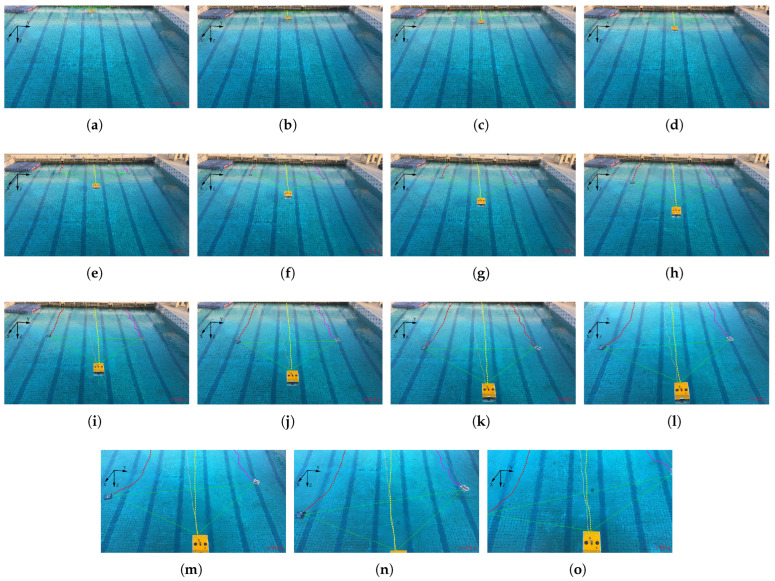
Sequential video screenshots of the multi-AUV formation target escort experiment (captured at t=0 s to t=70 s at 5-s intervals).

Figure 19a–o show sequential video snapshots of the formation at discrete time intervals: (a) t=0 s; (b) t=5 s; (c) t=10 s; (d) t=15 s; (e) t=20 s; (f) t=25 s; (g) t=30 s; (h) t=35 s; (i) t=40 s; (j) t=45 s; (k) t=50 s; (l) t=55 s; (m) t=60 s; (n) t=65 s; (o) t=70 s. In these figures, the yellow dotted line represents the movement trajectory of the escorted target, the red dotted line represents the movement trajectory of the AUV 2, the pink dotted line represents the movement trajectory of the AUV 3, and the green triangle represents the formation configuration at the current moment. These snapshots capture the entire formation process: (a) Initialization (t=0–10 s). The follower AUV quickly converge to the guard formation. (b) Steady escort (t=10–60 s). The triangular formation remains stable as the leader moves. (c) Late maneuver (t=60–70 s). Minor formation errors emerge, due to visual localization noise (e.g., underwater light scattering affecting camera accuracy). The relative distance between the AUVs deviates slightly from the desired values (maximum deviation of approximately 1 m). However, the overall triangular shape is preserved, indicating the robustness of the algorithm to mild sensor disturbances.

While there are minor discrepancies between the experimental and simulation results, primarily due to real-world uncertainties, such as sensor noise, and hardware dynamic delays, the system still achieves the three core objectives: (a) Precise formation maintenance. The triangular formation is maintained with an acceptable margin of error (≤1 m). (b) Reliable leader tracking. Followers accurately track the leader’s trajectory without divergence. (c) Collision safety. No inter-AUV collisions occurred throughout the experiment. These results provide strong experimental support for the practical application of the proposed multi-underwater vehicle formation cooperative escort technology in engineering scenarios.

## 7. Conclusions

This research represents significant progress in the field of multi-AUV formation cooperative escort for moving underwater targets. Regarding the algorithm design, we proposed a leader–follower based coordination escort algorithm to manage the overall movement of the multi-AUV formation. By using an undirected rigid graph based formation controller, the follower AUVs adjust their distances to the leader target according to the desired distance, thereby ensuring the formation’s integrity. This algorithm enhances the formation’s coordination ability in the face of communication-related challenges in the underwater environment. Regarding the path planning problem, we proposed an improved adaptive artificial potential field method is proposed to achieve formation obstacle avoidance. This method reliably enables the AUVs to avoid obstacles while approaching the goal. Simulation results and experiments demonstrated the excellent performance of the proposed algorithms. Physical experiments further highlighted the superiority of our proposed algorithms in terms of formation maintenance, target escorting, trajectory tracking, and obstacle avoidance. In terms of future research directions, there are several promising areas to further enhance the performance of, and expand the scope of application for, multi-AUV formation cooperative escort. For example, incorporating neural networks into the existing algorithms could further compensate for system uncertainties, reducing convergence time while simultaneously enhancing the system’s anti-interference capability. In the experimental verification, however, we only conducted the escort formation maintenance experiment. Next, we need to conduct experiments on movement planning and obstacle avoidance in a multi-obstacle environment.

## Data Availability

Due to privacy or ethical restrictions, data are unavailable.
